# A Simple and Rapid “Turn-On” Fluorescent Probe Based on Binuclear Schiff Base for Zn^2+^ and Its Application in Cell Imaging and Test Strips

**DOI:** 10.3390/molecules29245850

**Published:** 2024-12-11

**Authors:** Jinghui Cheng, Yi Li, Zhiye Zhu, Huijuan Guan, Jinsong Zhai, Yibing Xiang, Man Wang

**Affiliations:** 1 Key Laboratory of Chemo/Biosensing and Detection of Xuchang, Key Laboratory of Micro-Nano Materials for Energy Storage and Conversion of Henan Province, Henan Joint International Research Laboratory of Nanomaterials for Energy and Catalysis, College of Chemical and Materials Engineering, Xuchang University, Xuchang 461000, China; hunansheng202211@163.com (Y.L.); zzy_xcu@163.com (Z.Z.); guanhuijuan89@163.com (H.G.); 15939868518@163.com (J.Z.); 17527155029@163.com (Y.X.); 2 High & New Technology Research Center of Henan Academy of Sciences, No. 56 Hongzhuan Road, Zhengzhou 450002, China

**Keywords:** binuclear Schiff base, probe, test strip, cell imaging

## Abstract

A series of colorful binuclear Schiff bases derived from the different diamine bridges including 1,2- ethylenediamine (bis-Et-SA, bis-Et-4-NEt_2_, bis-Et-5-NO_2_, bis-Et-Naph), 1,2-phenylenediamine (bis-Ph-SA, bis-Ph-4-NEt_2_, bis-Ph-5-NO_2_, bis-Ph-Naph), dicyano-1,2-ethenediamine (bis-CN-SA, bis-CN-4-NEt_2_, bis-CN-5-NO_2_, bis-CN-Naph) have been designed and prepared. The optical properties of these binuclear Schiff base ligands were fully determined by UV–Vis absorption spectroscopy, fluorescence emission spectroscopy, and time-dependent-density functional theory (TD-DFT) calculations. The inclusion of D-A systems and/or π-extended systems in these binuclear Schiff base ligands not only enables adjustable RGB light absorption and emission spectra (300~700 nm) but also yields high fluorescence quantum efficiencies of up to 0.84 in MeCN solution. Then, with the ESIPT (excited-state intramolecular proton transfer) property, fluorescence analysis showed that the probe bis-Et-SA and bis-Ph-SA could recognize Zn^2+^ via the “turn on” mode in the MeCN solution. During the detection process, bis-Et-SA and bis-Ph-SA demonstrate rapid response and high selectivity upon the addition of Zn^2+^. The coordination of Zn^2+^ with the oxygen atom and Schiff base nitrogen atom in a tetrahedral geometry is confirmed by Job’s plot, FT-IR, and ^1^H NMR spectroscopy. In addition, the paper test and Hela cells were successfully carried out to detect Zn^2+^. Moreover, the sensitivity of bis-Et-SA and bis-Ph-SA is much better than that of those Schiff base ligands containing only one chelating unit [O^N^N^O].

## 1. Introduction

It is well known that metal ions play an essential role in living systems, both in growth and in metabolism. There is recognition and monitoring of the importance of metals in biological and environmental systems [1,2,3,4]. Zinc, a d-block element that does not undergo redox reactions, exhibits the second highest concentration in the hippocampus of the human brain, following iron. It is closely associated with enzyme regulation [5], neurotransmission [6,7], and apoptosis [8,9]. The disruption of zinc ion levels can impair the corresponding biochemical processes in the body, thereby contributing to the development of severe neurological disorders such as Alzheimer’s disease, Parkinson’s disease, epilepsy, ischemic stroke, and infantile diarrhea [10,11]. Therefore, it is imperative to investigate effective approaches for the recognition and monitoring of Zn^2+^ in environmental and biological specimens.

Despite the availability of various analytical methods for zinc determination, such as atomic absorption spectrometry, inductively coupled plasma-atomic emission spectrometry (ICP-AES), and inductively coupled plasma-mass spectrometry (ICP-MS) [12,13], imaging techniques, including fluorescence/phosphorescence [14,15,16,17,18,19], nuclear magnetic resonance imaging [20], mass spectrometry imaging [21], and photoacoustic imaging [22], have also been proposed for the purpose of detecting Zn^2+^. Among these methods, the utilization of fluorescent probes for Zn^2+^ detection has garnered increasing attention due to their remarkable sensitivity, exceptional selectivity, and operational convenience. Generally, a rational molecular design serves as an efficient strategy for developing functional organic fluorescent probes. However, the intricate and time-consuming synthesis-purification process hampers the practical application of organic fluorescent probes.

The Schiff base, belonging to a specific category of tetradentate [O^N^N^O] chelating ligands, can be easily synthesized through the condensation reaction between diamino compounds and salicylaldehyde derivatives [23,24,25]. Due to their facile preparation, moderate stability, diverse biological activities, rich photophysical properties, and strong coordination ability with metal ions, Schiff base ligands and their associated metal complexes have garnered significant attention in various fields, including catalysis [26,27,28], DNA cleavage [29,30], optical materials [31,32], magnetic materials [33,34], and supramolecular materials [35]. Additionally, Schiff base ligands featuring π-conjugated tetradentate [O^N^N^O] chelating systems exhibit the ability to coordinate with diverse metal ions, resulting in alterations of optical properties between the ligands and metal ions. Consequently, they have found extensive applications as optical probes for a wide range of metal ions, such as Zn^2+^ [36,37,38], Mg^2+^ [39,40], Cu^2+^ [41,42], Al^3+^ [36,43,44,45], La^3+^ [36], Pt^2+^ [46], F^−^ [45], and pH [47], and Hg^2+^ [45] optical probes based on Schiff base ligands have also been demonstrated. The primary challenge in metal ion detection lies in the selectivity of Schiff bases, as their tetradentate chelating properties enable them to coordinate with numerous metal ions and form stable complexes.

**Scheme 1:**
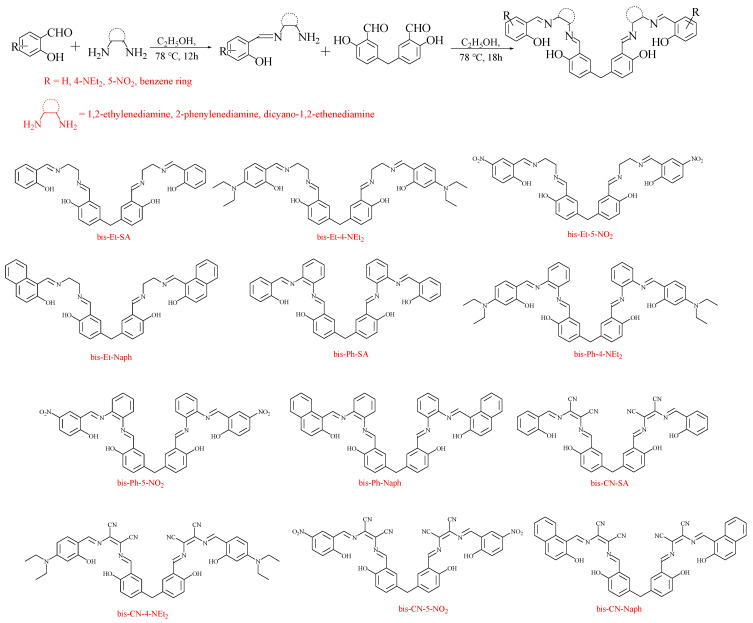
The synthetic route and chemical structures of binuclear Schiff base ligands in this work.

In this study, we systematically designed and synthesized novel binuclear Schiff base ligands featuring two tetradentate [O^N^N^O] chelating units. The salicylaldehyde moiety of these ligands incorporates electron-donating substituents, electron-accepting substituents, and/or π-extended systems (as depicted in Scheme 1). We thoroughly investigated the photophysical properties of this series of bis-Schiff base ligands with different bridges including 1,2-ethylenediamine (bis-Et-SA, bis-Et-4-NEt_2_, bis-Et-5-NO_2_, bis-Et-Naph), 1,2-phenylenediamine (bis-Ph-SA, bis-Ph-4-NEt_2_, bis-Ph-5-NO_2_, bis-Ph-Naph), and dicyano-1,2-ethenediamine (bis-CN-SA, bis-CN-4-NEt_2_, bis-CN-5-NO_2_, bis-CN-Naph) by employing UV–Vis absorption and fluorescent emission spectra as well as time-dependent density functional theory (TD-DFT) calculations. The incorporation of D–π–A systems and/or π–extended systems in these bis-Schiff base ligands not only ensures RGB light absorption and emission spectra (300~700 nm) but also yields high fluorescence quantum efficiencies of up to 0.84 in MeCN solution. Then, with the ESIPT (excited-state intramolecular proton transfer) [48,49] property, fluorescence analysis has shown that the probes bis-Et-SA and bis-Ph-SA could recognize Zn^2+^ via the “turn on” mode in a MeCN solution. During the detection process, bis-Et-SA and bis-Ph-SA demonstrate rapid response and high selectivity upon the addition of Zn^2+^. The coordination of Zn^2+^ with the oxygen atom and Schiff base nitrogen atom in a tetrahedral geometry is confirmed by Job’s plot, FT-IR, and ^1^H NMR spectroscopy. In addition, the paper test and Hela cells were successfully carried out to detect Zn^2+^. Moreover, the sensitivity of bis-Et-SA and bis-Ph-SA is much better than that of those containing only one chelating unit [O^N^N^O] Schiff base ligands.

## 2. Results and Discussion

### 2.1. Synthesis and Features of the Binuclear Schiff Base Ligands

The general method for preparing Schiff bases is straightforward and involves the condensation reaction of primary amines and aldehyde precursor, typically in an alcoholic solution or toluene under reflux conditions. Although the reaction is acid-catalyzed, catalysts are generally unnecessary when using aliphatic amines. To achieve high yields, water produced during the reversible reaction can be removed using a water knockout drum. For purification purposes, it is preferable to recrystallize Schiff bases (e.g., CH_2_Cl_2_/Et_2_O or CH_2_Cl_2_/hexane) rather than employing chromatography to avoid degradation through hydrolysis. Unlike many free Schiff bases that may not remain stable in solution and require template synthesis in the presence of a metal ion, most binuclear Schiff base ligands investigated in this study exhibit stability both in solution and in their solid state under ambient air conditions for synthesis, characterization, and application purposes. Therefore, binuclear Schiff base ligands containing two tetradentate [O^N^N^O] chelating units might be more suitable for sensing applications via coordination reactions. Thus, binuclear Schiff base ligands, including two tetradentate [O^N^N^O] chelating units, might be more suitable for sensing applications via coordination reaction. All the binuclear Schiff base ligands (Scheme 1) were prepared according to our previous report [50,51,52,53,54] and the reported literature [55,56].

### 2.2. Electronic Absorption Spectroscopy of the Binuclear Schiff Base Ligands

**Figure 1:**
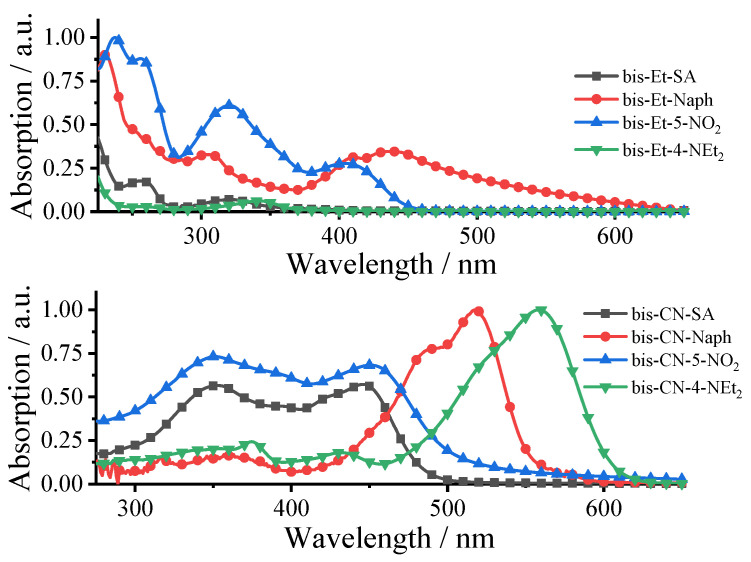
Normalized absorption spectra of the bis-Schiff ligands with the same bridges and different diethylamino-phenol in MeCN.

The UV–Vis absorption spectral data of all the binuclear Schiff base ligands are presented in Table 1 and Figure S1. The normalized UV–Vis absorption spectra of bis-Et-SA, bis-Et-4-NEt_2_, bis-Et-5-NO_2_, bis-Et-Naph, bis-CN-SA, bis-CN-4-NEt_2_, bis-CN-5-NO_2_, and bis-CN-Naph in MeCN are presented in Figure 1. Both bis-Et-SA and bis-Et-4NEt_2_ exhibit similar absorption bands centered at 250–260 nm, which are attributed to the π → π* transition involving molecular orbitals predominantly localized on the benzene ring and the C=N group. The absorption bands in the 330–340 nm region can be assigned to the *n* → π* transition involving molecular orbitals of the benzene ring and the C=N group. It is well-established that an increase in π-conjugation leads to a significant reduction in the energy gap between the highest occupied molecular orbital (HOMO) and the lowest unoccupied molecular orbital (LUMO), resulting in red-shifted absorption and emission spectra. This trend was observed for λ_abs_ with bis-Et-SA < bis-Et-4-NEt_2_ < bis-Et-5-NO_2_ < bis-Et-Naph., indicating that extending the π-conjugated system and introducing electron-donating or electron-withdrawing substituents into binuclear Schiff base ligands offers more efficient strategies for achieving red-shifted absorption spectra.

The bis-CN-4-NEt_2_ containing a D-A system, based on the π-conjugated backbone, notably facilitates intramolecular charge transfer, resulting in a maximum absorption band at 557 nm and red fluorescence (emission wavelength = 606 nm) with a high fluorescence quantum yield of up to 0.84 in MeCN. This verifies the effectiveness of the D-A system as a strategy for achieving red-shifted absorption bands.

**Figure 2:**
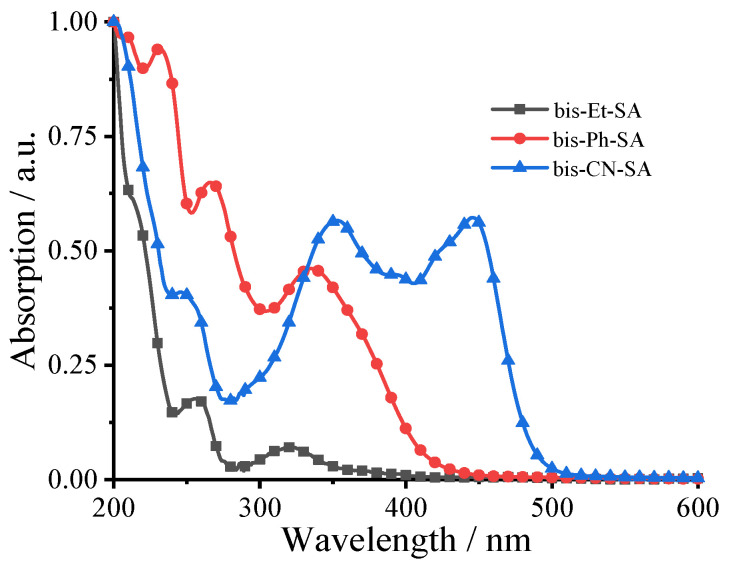
Normalized UV–Vis absorption spectra of the bis-Schiff ligands with the same diethylamino-phenol different bridges in MeCN.

The normalized UV–Vis absorption spectra of bis-Et-SA, bis-Ph-SA, and bis-CN-SA in MeCN are given in Figure 2. The absorption spectrum of bis-Ph-SA, which incorporates a π-conjugated bridge, exhibits a red shift compared to that of bis-Et-SA. By further extending the π-conjugated system, bis-CN-SA with a dicyano-1,2-ethenediamine π-conjugated bridge exhibits red-shifted absorption. The trend was observed in λ_abs_ with bis-Et-SA < bis-Ph-SA < bis-CN-SA. Colorful binuclear Schiff base ligands were demonstrated through meticulous modifications of their chemical structures.

### 2.3. Fluorescence Spectroscopy of the Binuclear Schiff Base Ligands

**Figure 3:**
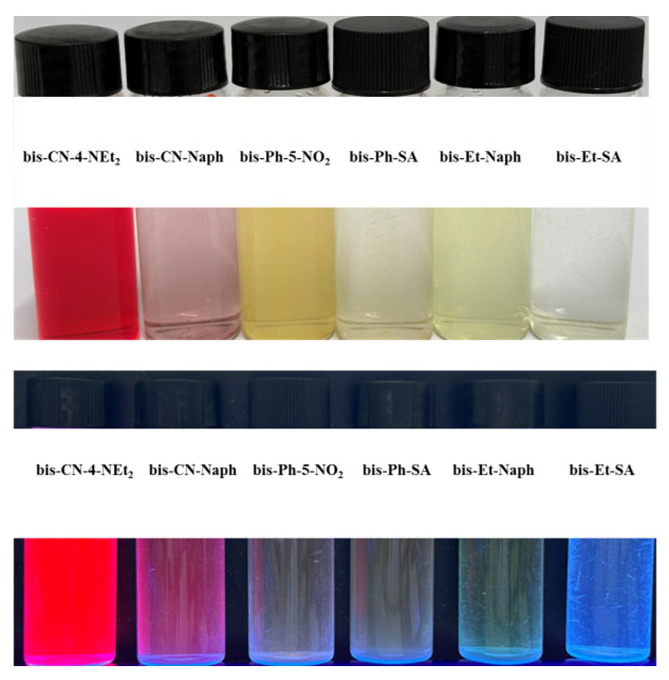
Images captured of the chosen binuclear Schiff base ligands in MeCN at ambient temperature. (Top: under natural sunlight; bottom: under 360 nm UV light).

The emission data of all the binuclear Schiff base ligands can be found in Table 1 and Figure S2. Under 360 nm UV light excitation, the binuclear Schiff base ligands in this study exhibit emissions of various colors, including red, pink, yellow, green, and blue, at room temperature, as depicted in Figure 3.

**Figure 4:**
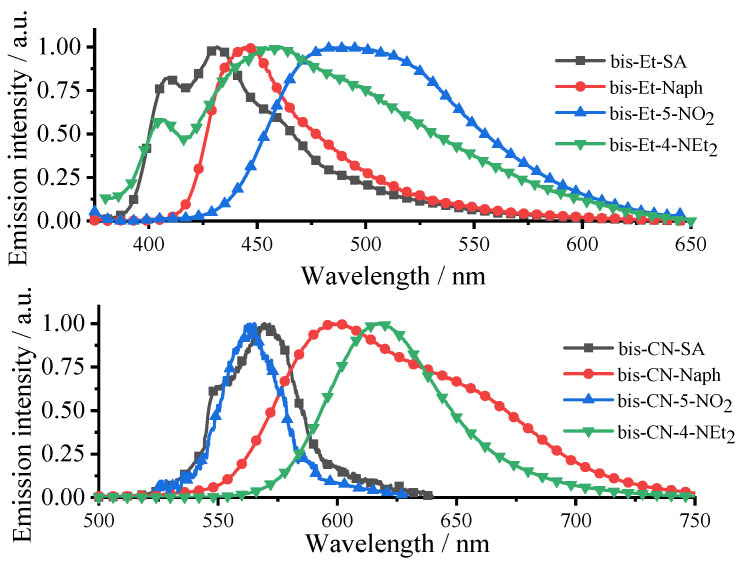
Normalized emission spectra were obtained for bis-Schiff ligands featuring the same bridges but varying diethylamino-phenol substituents in MeCN solvent.

The normalized fluorescence emission spectra of bis-Et-SA, bis-Et-4-NEt_2_, bis-Et-5-NO_2_, bis-Et-Naph, bis-CN-SA, bis-CN-4-NEt_2_, bis-CN-5-NO_2_, and bis-CN-Naph in MeCN are presented in Figure 4. These binuclear Schiff base ligands exhibit a broad fluorescence band ranging from 425 to 650 nm, primarily due to the π → π* transition. A trend can be observed in the λ_em_ values: bis-Et-SA < bis-Et-Naph < bis-Et-4-NEt_2_ < bis-Et-5-NO_2_ < bis-CN-SA ≈ bis-CN-5-NO_2_ < bis-CN-Naph < bis-CN-4-NEt_2_. This trend is consistent with the extension of the conjugated system and the introduction of electron-donating or electron-withdrawing substituents in these binuclear Schiff base ligands. However, it slightly deviates from the trend observed in their absorption spectra (Figure 1 and Figure 2). For bis-Et-SA and bis-Ph-SA, the maximum absorption wavelengths are observed at 325 nm and 336 nm, while the fluorescence emission peaks occur at 432 nm and 557 nm, respectively. Notably, a significant Stokes shift of 107 nm and 221 nm is observed for bis-Et-SA and bis-Ph-SA.

**Figure 5:**
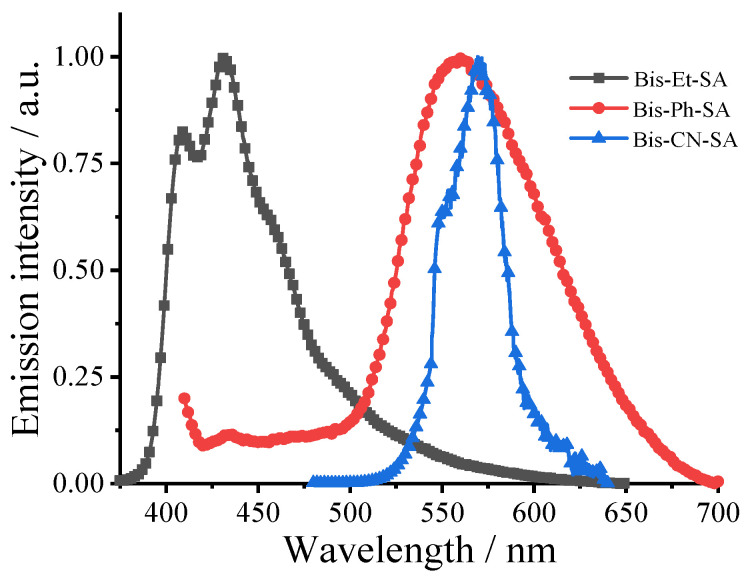
Normalized emission spectra were obtained for bis-Schiff ligands featuring various bridges and a consistent di-ethylamino-phenol in MeCN.

The normalized fluorescence emission spectra of bis-Et-SA, bis-Ph-SA, and bis-CN-SA in MeCN are presented in Figure 5. All three compounds contain the same diethylamino-phenol but differ in their bridges. Similar to the findings presented in Figure 2, a consistent trend was observed where the λ_em_ values decreased in the sequence of bis-Et-SA < bis-Ph-SA < bis-CN-SA. This can be attributed to the expansion of the π-conjugated system and the presence of donor-acceptor systems within these Schiff ligands.

The fluorescence quantum yields of these bis-Schiff ligands in solution were determined using the optical dilute method developed by Demas and Crosby [57]. A standard sample of quinine sulfate (Φr = 0.55, quinine dissolved in 0.05 mol dm^−3^ sulfuric acid) was used for comparison, and the results can be found in Table 1. It is worth noting that the bis-CN-4-NEt_2_ compound with a D-A system exhibited remarkably high fluorescence quantum yield, reaching up to 0.84. Consequently, when observed under normal room lighting conditions (Figure 3), the solution of bis-CN-4-NEt_2_ emitted a vibrant red color.

### 2.4. Electronic Structure Calculations of All the Binuclear Schiff Base Ligands

**Figure 6:**
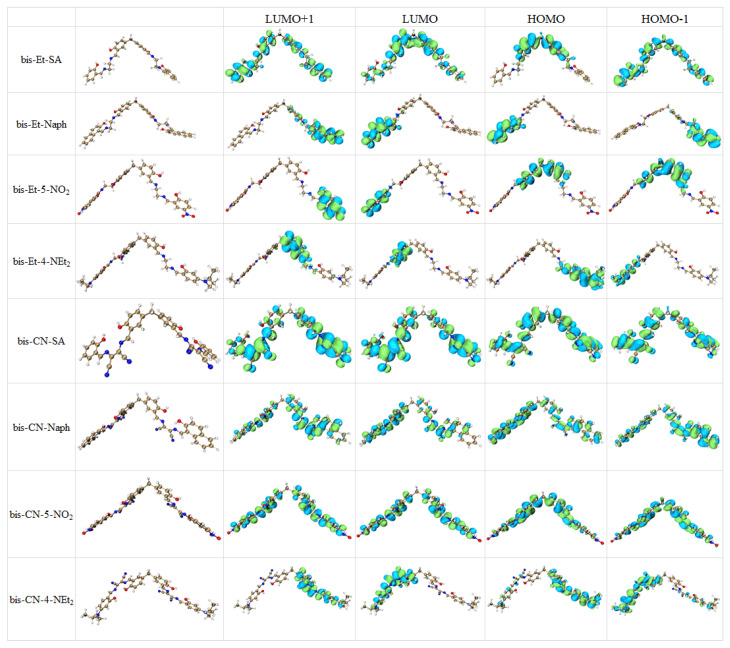
Frontier molecular orbitals for the bis-Schiff ligands with the different diethylamino-phenol and same bridges in MeCN at B3LYP 6-31G(d,p) level of theory.

**Figure 7:**
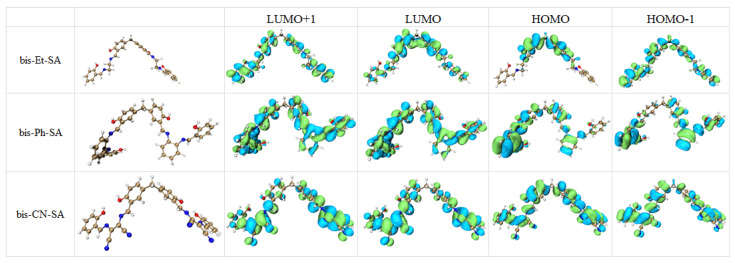
Frontier molecular orbitals for the bis-Schiff ligands with different bridges and the same diethylamino-phenol in MeCN at B3LYP 6-31G(d,p) level of theory.

To gain deeper insights into the excited states, we conducted TD-DFT calculations using ORCA 5.0.4 [58] for all bis-Schiff ligands. Cam-B3LYP/6-311G(d,p) was employed as the computational method. The PCM model with acetonitrile as the solvent was incorporated in all calculations. FMOs were obtained using Multiwfn 3.8 (dev) [59] and VMD 1.9.3 [60] software tools. To ensure that the optimized stationary point represented the energy minimum on the potential energy surface (PES), harmonic vibrational frequency analysis was performed at the same calculation level, resulting in no presence of imaginary vibrational frequencies. Theoretical modeling was carried out considering acetonitrile solvent conditions. Table 2 and Figure 6 and Figure 7 present diagrams illustrating LUMO+1, LUMO, HOMO, and HOMO-1 for ground states along with frontier molecular orbital energies of bis-Schiff ligands.

The optimized structures of bis-Et-SA, bis-Et-4-NEt_2_, bis-Et-5-NO_2_, bis-Et-Naph, bis-CN-SA, bis-CN-4-NEt_2_, bis-CN-5-NO_2_, and bis-CN-Naph are depicted in Figure 6. For compounds such as bis-Et-SA, bis-Et-4-NEt_2_, bis-Et-5-NO_2,_ and bis-Et-Naph that possess a non-conjugated bridge composed of 1,2 ethylenediamine units, it is observed that they exhibit similar LUMO levels (Table 2 and Figure 6), UV–Vis absorption spectra, and emission spectra. The introduction of electron-donating substituents (bis-Et-4-NEt_2_, −0.2524 eV) or electron-withdrawing substituents (bis-Et-5-NO_2_, −1.3098 eV) along with the extension of π-systems (bis Et-Naph, −0.5815 eV) affects the HOMO orbital energy. These findings agree well with experimental observations made on UV–Vis absorption and emission spectra.

Figure 7 illustrates the presence of bis-Et-SA, bis-Ph-SA, and bis-CN-SA in MeCN, which feature the same diethylamino-phenol but differ in terms of their bridges. Interestingly, the HOMO levels of bis-Et-SA, bis-Ph-SA, and bis-CN-SA containing diethylamino-phenol but differing in bridges were found to be approximately 7.4 eV. In contrast, the LUMO levels varied from −2.0949 to −0.2059 eV (Table 2), indicating that the bridges primarily affect the LUMO levels of these bis-Schiff ligands. A comprehensive understanding of how to control both HOMO and LUMO levels of Shiff ligands is crucial for fine-tuning absorption and emission bands.

### 2.5. Chemosensors Based on Bis-Schiff Base Ligands

**Figure 8:**
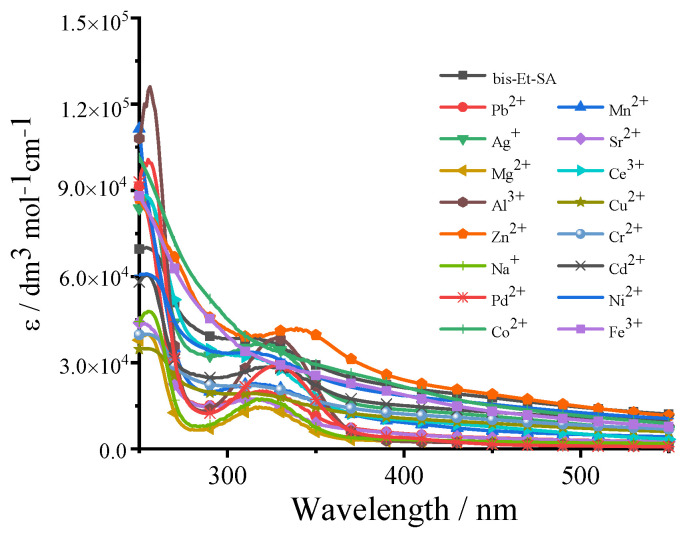
The UV–Vis absorption spectra of bis-Et-SA (1.0 × 10^−5^ mol dm^−3^ in MeCN) were observed upon the addition of 2 equiv. of different metal ions.

**Figure 9:**
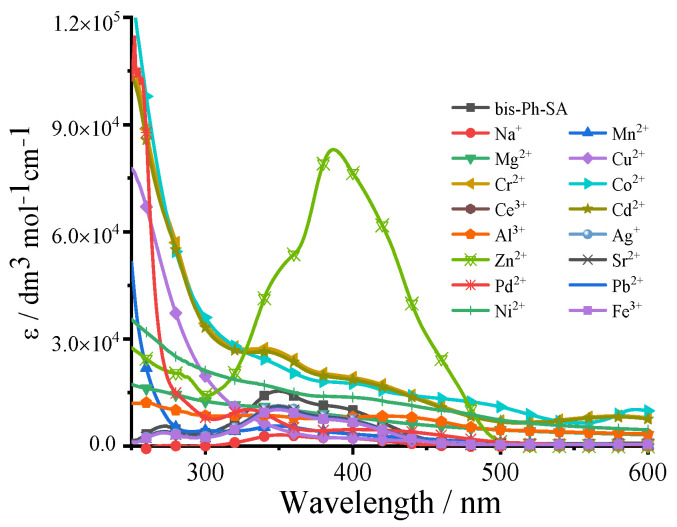
The UV–Vis absorption spectra of bis-Ph-SA (1.0 × 10^−5^ mol dm^−3^ in MeCN) were observed upon the addition of 2 equiv. of different metal ions.

Bis-Et-SA and bis-Ph-SA were employed as chemosensors. Both compounds demonstrated comparable sensing capabilities towards a range of metal ions, including Ag^+^, Al^3+^, Ce^3+^, Cd^2+^, Co^2+^, Cr^2+^, Cu^2+^, Fe^3+^, Mg^2+^, Mn^2+^, Na^+^, Ni^2+^, Pb^2+^, Pd^2+^, Sr^2+^, and Zn^2+^ was studied in MeCN. The optical response of bis-Et-SA and bis-Ph-SA toward various metal ions was studied by UV–Vis absorption spectroscopy, as shown in Figure 8 and Figure 9. Obviously, adding Zn^2+^ resulted in the absorption peak of bis-Et-SA redshifts from 317 nm to 341 nm (Figure 8). The UV–Vis absorption spectroscopy of bis-Ph-SA is more obvious than that of bis-Et-SA when adding Zn^2+^. The intensity of the absorption peak at 400 nm in the UV–Vis absorption spectrum of bis-Ph-SA exhibited a significant enhancement upon the introduction of Zn^2+^ (Figure 9). A similar response phenomenon was observed in bis-Et-4-NEt_2_ (Figure S4) and bis-Et-Naph (Figure S5). Therefore, bis-Et-SA, bis-Et-4-NEt_2_, bis-Et-Naph, and bis-Ph-SA have the potential to be used as simple and rapid probes for detecting Zn^2+^.

**Figure 10:**
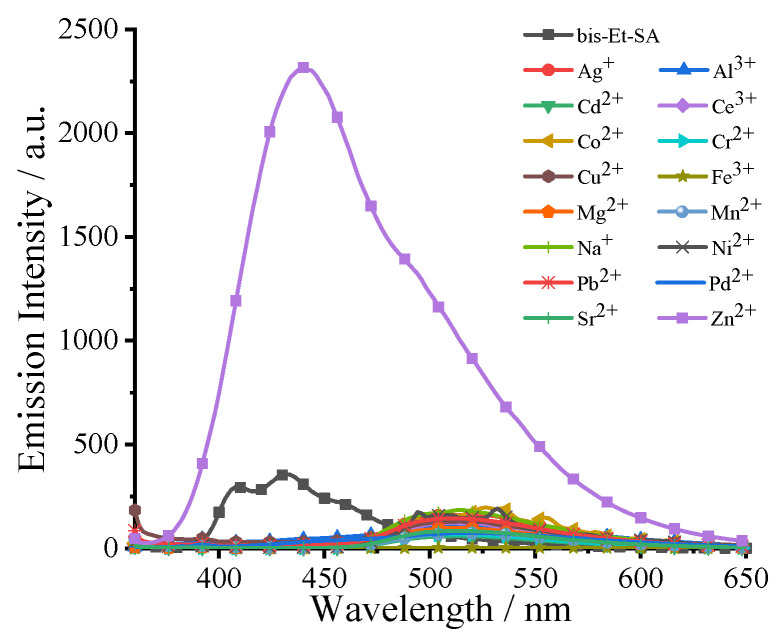
The emission spectra of bis-Et-SA (1.0 × 10^−5^ mol dm^−3^ in MeCN, excited at 360 nm) upon the addition of 2 equiv. of different metal ions.

**Figure 11:**
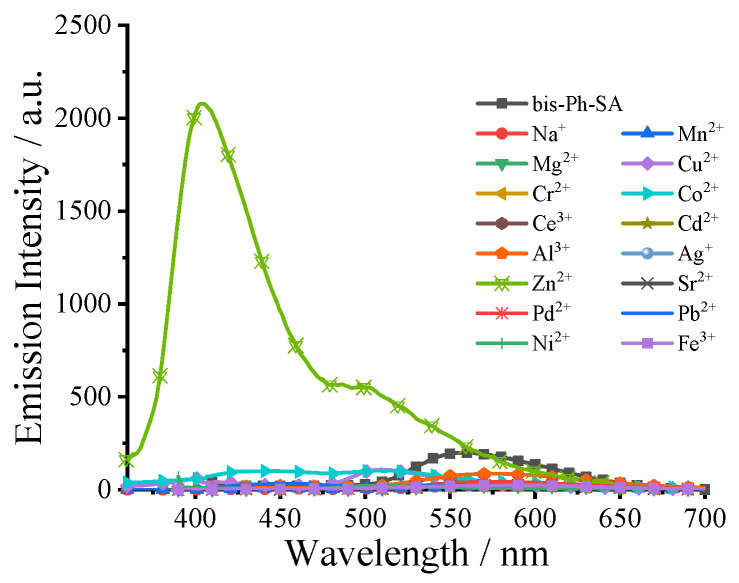
The emission spectra of bis-Ph-SA (1.0 × 10^−5^ mol dm^−3^ in MeCN, excited at 340 nm) were observed upon the addition of 2 equiv of various metal ions.

The selectivity is undoubtedly a crucial characteristic of a probe, referring to its preferential response towards the primary ion compared to other ions existing in the solution. The selectivity of fluorescence detection is better than that of colorimetric detection for bis-Et-SA and bis-Ph-SA, as demonstrated in Figure 10 and Figure 11. The addition of various metal ions to bis-Et-SA resulted in little changes in the emission intensity at 450 nm. However, upon the addition of Zn^2+^, there was a significant increase in the emission intensity at 450 nm by approximately 10-fold, indicating the excellent selectivity of bis-Et-SA for detecting Zn^2+^. Meanwhile, for bis-Ph-SA, adding many other metal ions led to no or a little enhancement of emission intensity at 575 nm; but, when Zn^2+^ was added, the emission peak blue shifted to 405 nm and increased sharply to over 2000 fold, indicating that bis-Ph-SA had a good selectivity to detect Zn^2+^. A similar fluorescence phenomenon was observed in bis-Et-4-NEt_2_ (Figure S6) and bis-Et-Naph (Figure S7).

**Figure 12:**
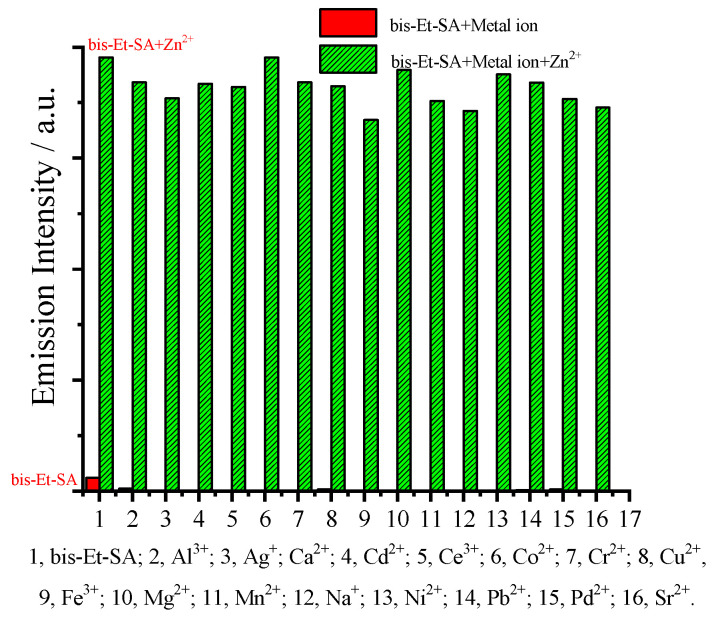
The selectivity of bis-Et-SA (1.0 × 10^−5^ mol dm^−3^ in MeCN, with emission measured at 450 nm and excited at 360 nm) towards 2.0 equiv. of other metal ions, and Zn^2+^ was investigated.

**Figure 13:**
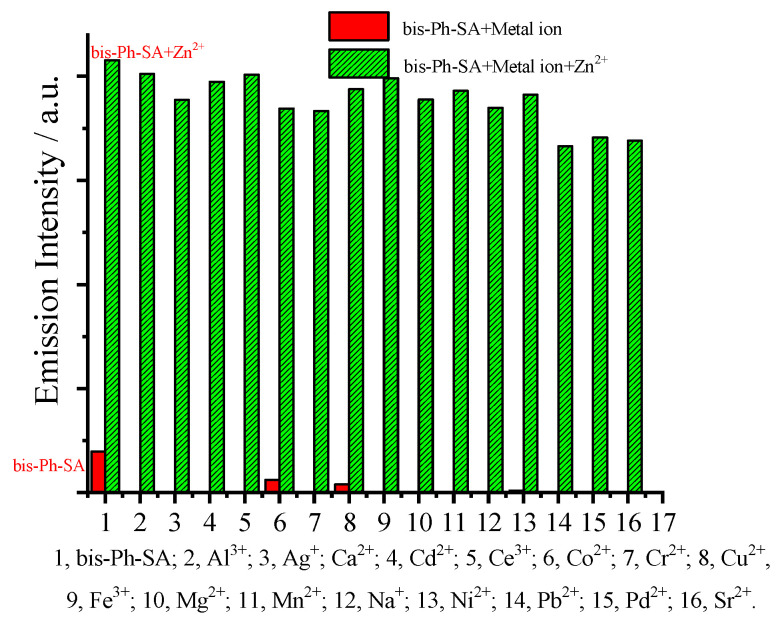
Selectivity of bis-Ph-SA (1.0 × 10^−5^ mol dm^−3^ in MeCN, emission measured at 405 nm and excited at 340 nm) toward 2.0 equiv. of other metal ions and Zn^2+^.

**Figure 14:**
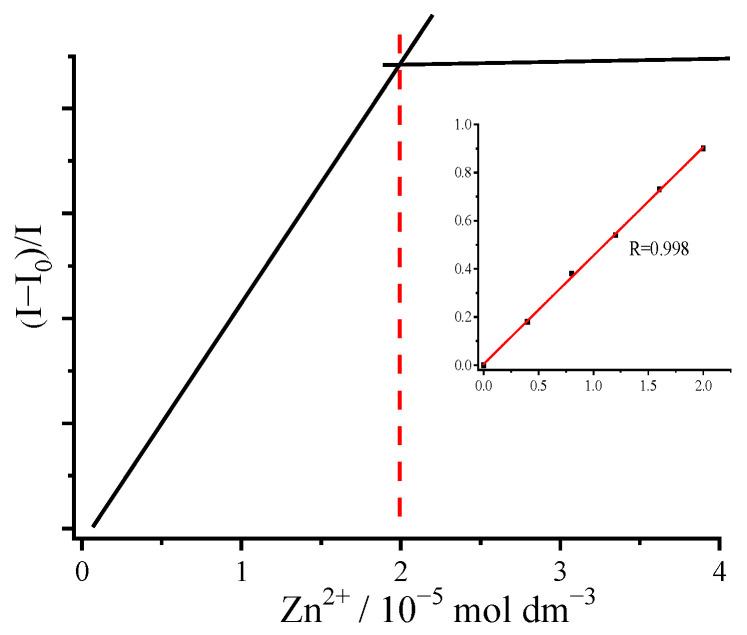
Plot of (I − I0)/I (emission at 450 nm) as a function of Zn^2+^ concentration based on bis-Et-SA (1.0 × 10^−5^ mol dm^−3^ in MeCN).

**Figure 15:**
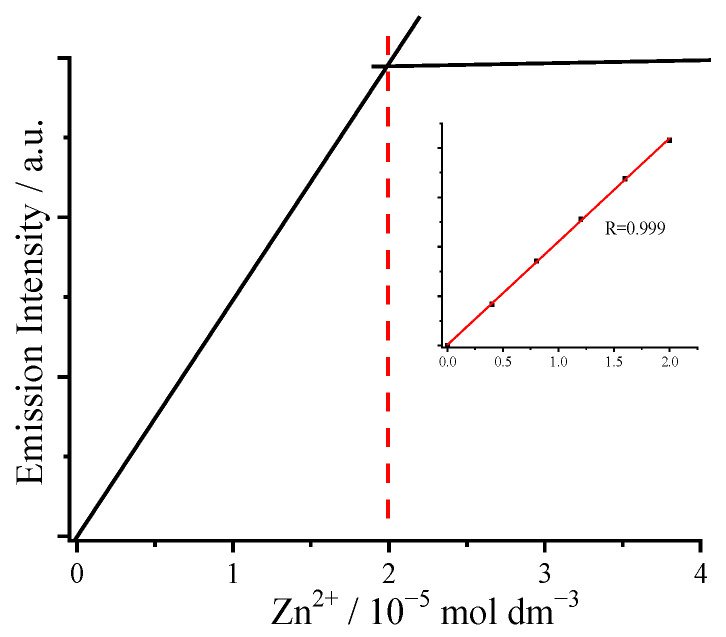
Plot of emission intensity at 405 nm as a function of Zn^2+^ concentration based on bis-Ph-SA (1.0 × 10^−5^ mol dm^−3^ in MeCN).

In addition, competition experiments were also conducted in the presence of bis-Et-SA and bis-Ph-SA, along with a mixture of 2 equiv. of Zn^2+^ and 2 equiv. of the other metal ions (Figure 12 and Figure 13) to investigate the practical applicability of bis-Et-SA and bis-Ph-SA as selective fluorescence chemosensors for Zn^2+^. The fluorescence intensity of solutions containing both Zn^2+^ and the other metal ions exhibits no discernible variation compared to those containing Zn^2+^ alone. The combined findings demonstrate that bis-Et-SA and bis-Ph-SA exhibit exceptional selectivity towards Zn^2+^ ions, displaying minimal interference from other metal ions. Consequently, they serve as highly efficient fluorescence chemosensors for Zn^2+^ detection. The fluorescence intensity at 450 nm for bis-Et-SA and 405 nm for bis-Ph-SA exhibited a gradual increase upon the addition of 0–2 equiv. of Zn^2+^ and saturation upon the addition of 2.0–4.0 equiv. of Zn^2+^ to the solution of bis-Et-SA and bis-Ph-SA (Figure 14 and Figure 15). The limits of detection (LODs) for bis-Et-SA and bis-Ph-SA, as defined by IUPAC (CDL = 3 Sb/m), were determined to be 1.32 ppm and 1.86 ppm, respectively, based on the analysis of 10 blank solutions.

### 2.6. Sensing Mechanism of Chemosensors Based on Bis-Schiff Base Ligands

**Figure 16:**
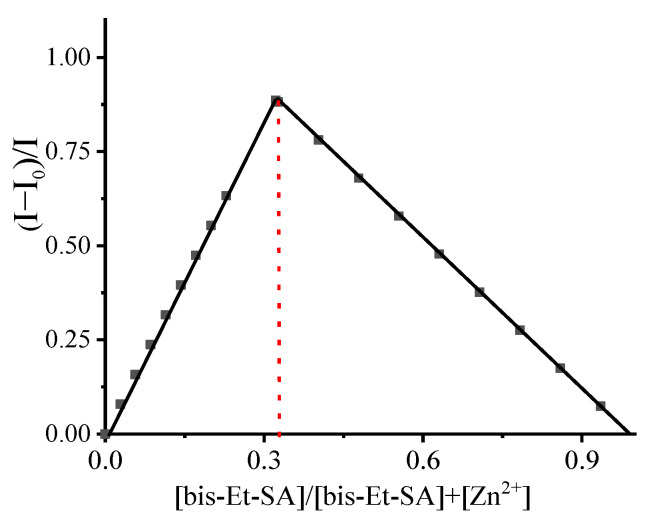
Job’s plot of bis-Et-SA and Zn^2+^ in CH_3_CN solution. The total concentration of bis-Et-SA and Zn^2+^ was 10 μM. The emission intensity at 450 nm.

**Figure 17:**
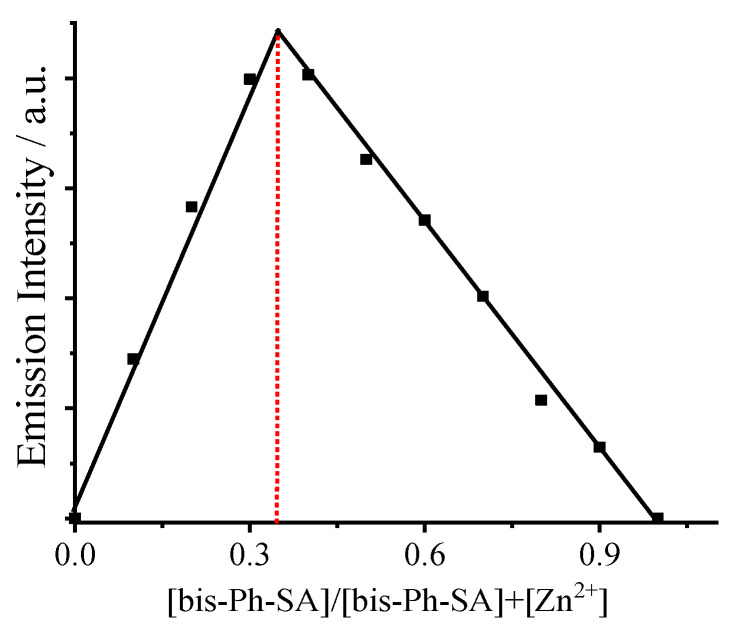
Job’s plot of bis-Ph-SA and Zn^2+^ in CH_3_CN solution. The total concentration of bis-Ph-SA and Zn^2+^ was 10 μM. The emission intensity at 405 nm.

The selectivity of these binuclear Schiff base ligands is much better than that of containing only one chelating unit [O^N^N^O] Schiff base ligands. The changes in fluorescence and UV–Vis absorbance spectra induced by Zn^2+^ ions are likely attributed to the coordination between binuclear Schiff base ligands and Zn^2+^ ions. The chelation effect will prevent the ESIPT and nonradioactive processes, thereby inducing an enhanced fluorescence phenomenon and a shift in emission wavelength. Job’s plot analysis reveals a 1:2 stoichiometric binding between bis-Et-SA, bis-Ph-SA, and Zn^2+^ ions (The dotted line in red is shown in Figure 14, Figure 15, Figure 16 and Figure 17).

**Figure 18:**
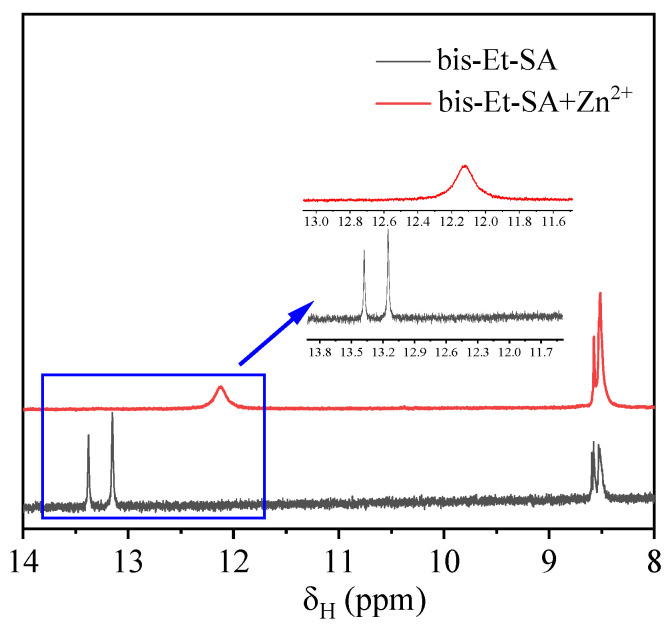
The fraction ^1^HNMR spectroscopy of bis-Et-SA and bis-Et-SA+Zn^2+^ in DMSO-d_6_.

The binding mode is also proved by the ^1^H NMR spectra. The hydroxy proton, located at approximately 13.37 ppm, can be attributed to the presence of -OH groups. Similarly, the proton signal at 13.15 ppm corresponds to the -CH=N- proton (Figure 18 and Figure S7). Due to more intramolecular hydrogen bonds, the chemical shift of -CH=N- is at 13.15 ppm. (Figure S8). Upon the addition of 2.0 eq of Zn^2+^ ions, the disappearance of the proton signals at 13.37 indicates deprotonation of the –OH groups and their involvement in coordination. The proton peaks at 13.15 ppm (-CH=N-) were significantly broadened and downfield shifted to 11.95 ppm, which indicates the participation of nitrogen atoms in coordination with Zn^2+^ ions.

**Figure 19:**
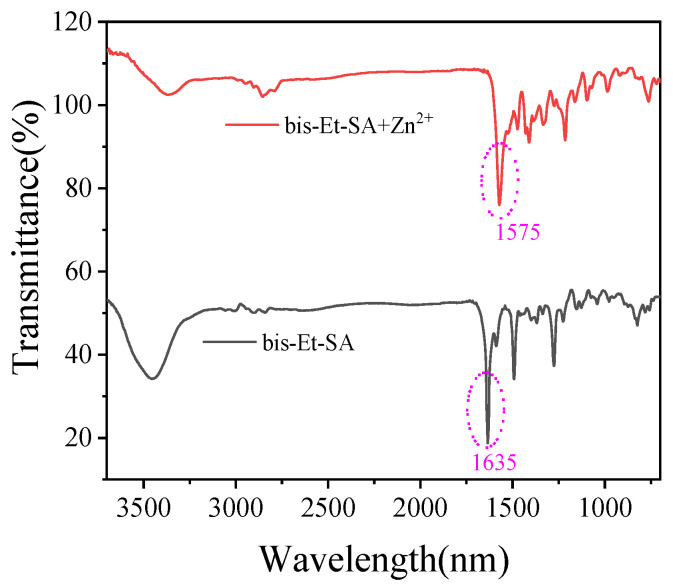
The IR spectra of bis-Et-SA and bis-Et-SA+Zn^2+^.

**Figure 20:**
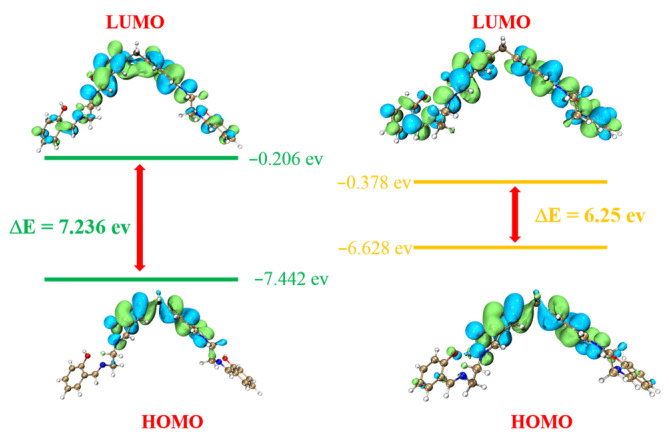
Energy level diagram for the frontier molecular orbitals of bis-Et-SA (**left**) and its zinc complex (**right**) calculated by using the B3LYP/6–31G basis set.

**Figure 21:**
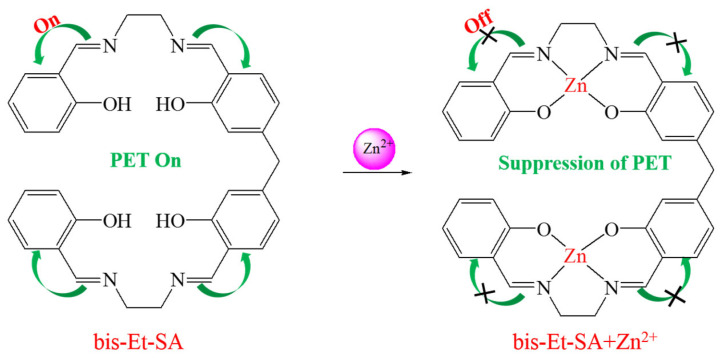
The proposed binding mechanism of bis-Et-SA with Zn^2+^.

The FT-IR spectra of bis-Et-SA and bis-Et-SA+Zn^2+^ were examined. As depicted in Figure 19, a noticeable shift in the stretching vibration absorption of the C=N bond was observed, shifting from 1635 cm^−1^ (free bis-Et-SA) to 1575 cm^−1^ (bis-Et-SA+Zn^2+^). The coordination of Zn^2+^ was observed with the nitrogen in the C=N band, as evidenced by the ^1^H NMR and FT-IR data. The calculated theoretical results suggest a reasonable reduction in the energy gap between HOMO and LUMO for the bis-Et-SA+Zn^2+^ compared to that of bis-Et-SA (Figure 20), which agree with the observed changes in absorption spectra upon addition of Zn^2+^ ions. The proposed mechanism illustrating the formation of the complex between bis-Et-SA and Zn^2+^ ion can be seen in Figure 21. The lack of light emission from bis-Et-SA in acetonitrile solution is primarily attributed to the photoinduced electron transfer mechanism (PET), while the enhanced fluorescence upon the addition of zinc ions can be ascribed to the constraints imposed by the suppression of PET.

### 2.7. Fluorescence Imaging of Zn^2+^ Chemosensors Based on Bis-Schiff Base Ligands in Living Cells

**Figure 22:**
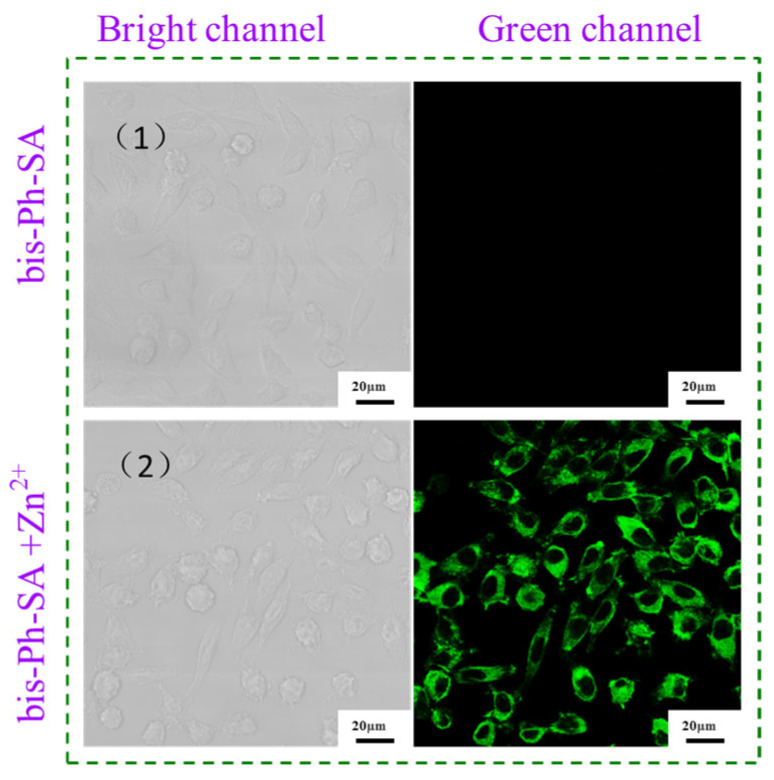
Fluorescent images were captured of Hela cells. (1) Bis-Ph-SA (30 μM) was added to the culture medium and incubated with Hela cells for 40 min. (2) Prior to treatment, Hela cells were exposed to bis-Ph-SA (30 μM) for 40 min, followed by an additional 40 min incubation with Zn^2+^ ions (60 μM).

Considering their excellent sensing properties towards Zn^2+^ ions, fluorescence imaging experiments were conducted to further investigate the capability of bis-Et-SA and bis-Ph-SA in detecting Zn^2+^ ions within living biological cells. The bis-Et-SA, which exhibits poor solubility in DMSO-H_2_O (1:9), demonstrates limited cellular uptake within Hela cells in living biological systems. Only bis-Ph-SA has been successfully used to image in Hela cells. As depicted, minimal fluorescence was observed in the green channel of Hela cells following incubation with bis-Ph-SA (30 μM) for 40 min (Figure 22). However, upon addition and subsequent incubation of Zn^2+^ ions (60 μM) for 40 min, a robust green emission in the green channel was detected due to the coordination between bis-Ph-SA and Zn^2+^ (Figure 22). These findings revealed that bis-Ph-SA can be used as a fluorescent probe for real-time monitoring of intracellular Zn^2+^ levels.

**Figure 23:**
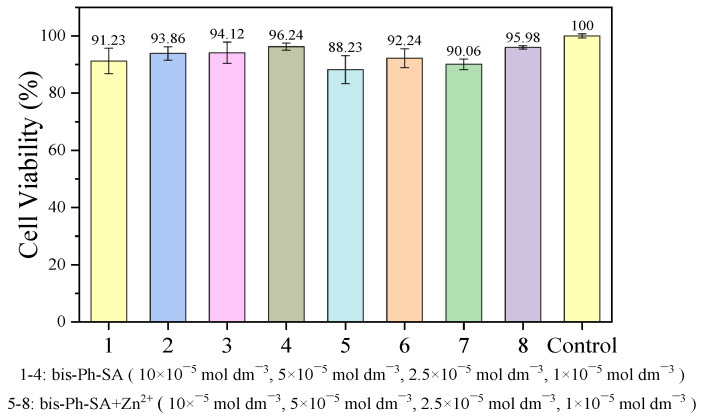
The cytotoxicity test of bis-Ph-SA.

It is of high importance that the fluorescent probes have good biocompatibility with low toxicity to target bio-substrates. The metabolic viability of Mouse lung fibroblasts (L929) after incubation with bis-Ph-SA was determined through a Cell Counting Kit-8 (CCK-8) assay to evaluate the cytotoxicity of bis-Ph-SA. Samples of 30 μM with different concentrations (1, 2.5, 5, 10 × 10^−5^ mol dm^−3^) of bis-Ph-SA were added into each well after the cells were seeded at a density of 2000 per well in a 96-well plate. Meanwhile, 30 μM samples with different concentrations (1, 2.5, 5, 10 × 10^−5^ mol dm^−3^) of bis-Ph-SA and 60 μM Zn^2+^ ions were added into each well after the cells were seeded at a density of 2000 per well in a 96-well plate. Thereafter, the CCK-8 assay was used for 24 h, and the OD value was measured by Eliasa at a wavelength of 450 nm. The results suggest that the cell viability remains about 90% after a 24 h incubation with either bis-Ph-SA or bis-Ph-SA+Zn^2+^ (Figure 23), indicating that bis-Ph-SA has low cytotoxicity during the test period.

### 2.8. Test Papers Detection of Zn^2+^ Based on Bis-Schiff Base Ligands

**Figure 24:**
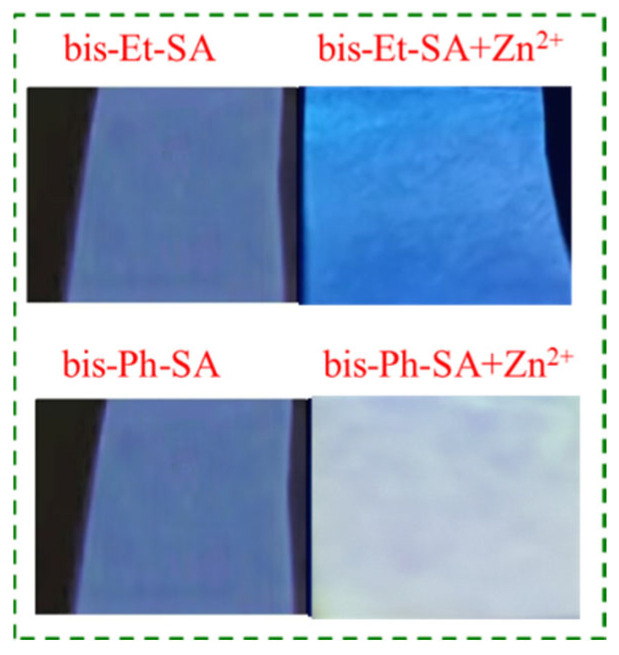
Pictures of bis-Et-SA and bis-Ph-SA test strips treated with Zn^2+^. The photographs were captured upon exposure to 360 nm UV light.

The fact that bis-Et-SA and bis-Ph-SA have good selectivity and rapid responsiveness to Zn^2+^ has motivated us to investigate the feasibility of developing solid-state materials (test papers) for rapid detection applications. Initially, we prepared test papers by cutting filter papers and impregnating them with solutions containing 1 mM bis-Et-SA and bis-Ph-SA in CH_3_CN. After solvent evaporation, the dried test papers were exposed to a solution of Zn^2+^ followed by observation under a 360 nm UV lamp to monitor changes in fluorescence color. Figure 24 illustrates that no fluorescence was detected when the test strips were loaded with dried solutions of bis-Et-SA and bis-Ph-SA. However, upon the addition of Zn^2+^, the fluorescence intensity gradually transitioned from non-existent to blue for bis-Et-SA, while yellow–green fluorescence was observed for bis-Ph-SA.

## 3. Materials and Methods

### 3.1. Materials and Instrumentation

The reagents were procured from commercial suppliers and utilized without any additional purification. The compound 5,5′-Methylene-bis-salicylaldehyd was synthesized according to the literature [50]. The ligands were synthesized following established protocols as described in the previous literature [51,52,53,54,55,56]. ^1^HNMR spectra (400 MHz) were recorded in CDCl_3_ or DMSO-d_6_. The chemical shifts were reported in ppm using tetramethylsilane as the internal standard. UV–vis absorption spectra were measured using a Cary 5000 (Agilent, Santa Clara, CA, USA) spectrophotometer with quartz cuvettes having a path length of 1 cm. Fluorescence spectra were acquired at room temperature using an F-4600 Fluorescence spectrophotometer (Hitachi, Tokyo, Japan). Both excitation and emission had a slit width of 5.0 nm, while the photon multiplier voltage was set to 400 V. Samples in solution and powder form were placed in quartz cuvettes with a path length of 10.0 mm (volume: 3.5 mL) and quartz tubes, respectively. The melting point was measured with a microscopic melting point apparatus (SGW, X-4A, Shanghai, China).

### 3.2. Measurement of Fluorescence Quantum Yield (Φ)

The Fluorescence Quantum Yield was determined using the optical dilution method developed by Demas and Crosby [57]. A standard solution of quinine sulfate (with a quantum yield of 0.55 in 0.05 mol dm^−3^ sulfuric acid) was used for reference calculations. The calculation formula employed is as follows: Φs = Φ_r_(B_r_/B_s_)(n_s_/n_r_)^2^(D_s_/D_r_), where the subscripts s and r denote the sample and reference solutions, respectively; n represents the refractive index of the solvents; D corresponds to the integrated intensity. The excitation intensity B can be calculated using B = 1 − 10^–A L^, where A refers to the absorbance at the excitation wavelength, and L denotes the optical path length (L = 1 cm for all cases). The refractive indices of the solvents were obtained from reliable sources at room temperature. An estimated error margin of ±10% was considered when determining Φ values.

### 3.3. Computational Details

All the calculations were performed with ORCA 5.0.4 [58]. Cam-B3LYP/6-311G(d,p) was used. All the calculations introduced the PCM model with acetonitrile as the solvent. Multiwfn 3.8 (dev) [59] and VMD 1.9.3 [60] were used to obtain the FMOs. To ensure that the fully optimized stationary point was the energy minima of the potential energy surface (PES), harmonic vibrational frequency analysis was performed at the same calculation level, and no imaginary vibrational frequencies were presented.

### 3.4. Measurement of Metal Ion Sensing

The titration experiments for each metal ion were initiated using a binuclear Schiff base ligand (3 mL) with a known concentration of 1.0 × 10^−5^ mol dm^−3^ in MeCN. Metal salts (1.0 × 10^−4^ mol dm^−3^ in H_2_O) of various types were employed for the titration process. All fluorescence measurements were recorded for at least 15 min following the addition of the metal salt to the solutions containing binuclear Schiff base ligands while maintaining room temperature.

### 3.5. Cell Culture Methods and Imaging

The imaging of Hela cells was conducted using the Fluorescence Vertical Microscope Leica STELLARIS 5 (Leica, Wetzlar, Germany). Hela cells were cultured in DMEM, a modified eagle medium, supplemented with 10% fetal bovine serum, penicillin (100 units mL^−1^), and streptomycin (100 mg mL^−1^), and maintained at a temperature of 37 °C with a CO_2_ concentration of 5%. Following removal of the incubation media and three rinses with PBS, the cells were exposed to a dye solution (30 μM) in PBS for a duration of 40 min at room temperature. Subsequently, the cells underwent three washes with PBS before being treated with Zn^2+^ ions (60 μM) for 40 min. Finally, confocal microscopy was employed to visualize the cellular structures.

Cytotoxicity of L929 was performed by immersion in the 10% PBS DMEM and holding it for 24 h in 5% CO_2_ at 37 °C. L929 cells were seeded in 96-multiwell plates in 500 μL 10% PBS DMEM and kept running for 24 h at 37 °C. Then, the old culture medium was removed. Samples of 30 μM with different concentrations (1, 2.5, 5, 10 × 10^−5^ mol dm^−3^) of bis-Ph-SA were added into each well; meanwhile, samples of 30 μM with different concentrations (1, 2.5, 5, 10 × 10^−5^ mol dm^−3^) of bis-Ph-SA and 60 μM Zn^2+^ ions were added into each well, after the cells were seeded at a density of 2000 per well in the 96-well plate and kept running for 24 h at 37 °C in the presence of 5% CO2. Then, a CCK-8 assay was performed to evaluate cell viability [61,62,63].

$$Cells\;viability\left(\%\right)=\frac{{OD}_{treated}}{{OD}_{control}}\times 100\%$$

## 4. Conclusions

We have systematically synthesized and investigated the photophysical properties of a series of binuclear Schiff base ligands, incorporating diverse diamine bridges, electron-accepting substituents, electron-donating substituents, D-A systems, and/or π-extended systems. This has resulted in adjustable RGB light absorption and emission characteristics. The experimental results and TD-DFT calculations have convincingly demonstrated the ability of D-A systems and π-extended systems to effectively reduce the energy gap, resulting in a red shift in both absorption and emission bands. Furthermore, bis-Et-SA and bis-Ph-SA were employed for the selective detection of Zn^2+^ simultaneously, exhibiting distinct emission wavelengths and discernible colors when excited in MeCN. The bis-Et-SA and bis-Ph-SA exhibited remarkable selectivity and a low limit of detection towards Zn^2+^. Furthermore, the binding modes of bis-Et-SA and bis-Ph-SA with Zn^2+^ were investigated using Job’s plot, FT-IR, and ^1^H NMR techniques. The test paper loaded with these ligands enabled rapid visual detection of Zn^2+^. Fluorescence imaging experiments demonstrated the capability of bis-Ph-SA to detect Zn^2+^ ions in Hela cells, suggesting its promising application for tracking Zn^2+^ in biological systems. Ongoing research in our laboratory focuses on exploring the potential applications of these colorful binuclear Shiff base ligands.

## Figures and Tables

**Table 1:** Photophysical data of all the binuclear Schiff base ligands at room temperature.

	Medium	λ_abs_/nm ε/dm^3^ mol^−1^ cm^−1^)	λ/nm	Φ
bis-CN-SA	MeCN	352 (3.38 × 10^4^); 446 (3.42 × 10^4^)	570	0.09
bis-CN-5-NO_2_	MeCN	348 (3.83 × 10^4^); 449 (3.60 × 10^4^)	563	0.29
bis-CN-Naph	MeCN	517 (5.71 × 10^3^)	597	0.38
bis-CN-4-NEt_2_	MeCN	373 (1.01 × 10^4^); 430 (7.47 × 10^3^); 557 (4.27 × 10^4^)	616	0.84
bis-Ph-SA	MeCN	266 (7.10 × 10^4^); 336 (5.03 × 10^4^)	557	0.25
bis-Ph-5-NO_2_	MeCN	326 (3.20 × 10^4^); 506 (1.23 × 10^4^)	588	0.11
bis-Ph-Naph	MeCN	338 (5.13 × 10^4^); 445 (1.75 × 10^4^); 470 (1.55 × 10^4^)	537	0.13
bis-Ph-4-NEt_2_	MeCN	343 (1.08 × 10^4^); 385 (8.68 × 10^3^)	597	0.08
bis-Et-SA	MeCN	258 (1.01 × 10^3^); 325 (5.69 × 10^3^)	432	0.30
bis-Et-5-NO_2_	MeCN	320 (6.38 × 10^4^); 406 (2.94 × 10^4^)	487	0.10
bis-Et-Naph	MeCN	309 (1.84 × 10^4^); 409 (1.77 × 10^4^); 438 (1.95 × 10^4^)	444	0.27
bis-Et-4-NEt_2_	MeCN	284 (4.72 × 10^3^); 342 (1.19 × 10^4^)	461	0.12

Quantum yield standard (Φ_r_): quinine sulfate (Φ_r_ = 0.55, quinine in 0.05 mol dm^−3^ sulfuric acid).

**Table 2:** The energy gap and electron volts (eV) of the frontier molecular orbitals in the bis-Schiff ligands.

	HOMO-1/eV	HOMO/eV	LUMO/eV	LUMO+1/eV	Energy Gap/eV
bis-Et-SA	−7.7196	−7.4423	−0.2059	−0.1123	7.2364
bis-Et-4-NEt_2_	−6.6918	−6.6719	−0.2524	−0.0785	6.4195
bis-Et-5-NO_2_	−7.7115	−7.3722	−1.3098	−1.3006	6.0624
bis-Et-Naph	−7.2827	−7.2003	−0.5815	−0.4640	6.6188
bis-Ph-SA	−7.2636	−7.2225	−0.6966	−0.6657	6.5259
bis-Ph-4-NEt_2_	−6.6793	−6.6546	−0.7126	−0.5331	5.9420
bis-Ph-5-NO_2_	−7.3669	−7.2971	−1.3421	−1.3321	5.9550
bis-Ph-Naph	−7.1415	−7.0589	−0.9104	−0.8172	6.1485
bis-CN-SA	−7.5245	−7.4222	−2.0949	−2.0700	5.3273
bis-CN-4-NEt_2_	−6.7707	−6.7577	−1.9907	−1.8933	4.7670
bis-CN-5-NO_2_	−7.6977	−7.5406	−2.2189	−2.1886	5.3217
bis-CN-Naph	−7.2273	−7.1938	−2.1852	−2.1807	5.0086

## Data Availability

The data presented in this study are available on request from the corresponding author.

## Supplementary Materials

The following supporting information can be downloaded at https://www.mdpi.com/article/10.3390/molecules29245850/s1, the synthesis data, the NMR data, and the mass spectrometry data of all the binuclear Schiff bases are in the Supporting Materials. Figure S1: Absorption spectra of all binuclear Schiff base ligands in MeCN at room temperature; Figure S2: Normalized emission spectra of all the bis-Schiff ligands in MeCN at room temperature. Figure S3: Absorption spectra of bis-Et-4-NEt_2_ (1.0 × 10^−5^ mol dm^−3^ in MeCN) upon the addition of 2 equiv. of different metal ions. Figure S4: Absorption spectra of bis-Et-Naph (1.0 × 10^−5^ mol dm^−3^ in MeCN) upon the addition of 2 equiv. of different metal ions. Figure S5: The emission spectra of bis-Et-4-NEt_2_ (1.0 × 10^−5^ mol dm^−3^ in MeCN, excited at 370 nm) upon the addition of 2 equiv. of different metal ions. Figure S6: The emission spectra of bis-Et-Naph (1.0 × 10^−5^ mol dm^−3^ in MeCN, excited at 360 nm) upon the addition of 2 equiv. of different metal ions. Figure S7: The ^1^HNMR spectroscopy of bis-Et-SA and bis-Et-SA+Zn^2+^ in DMSO-d_6_ (Top: bis-Et-SA. Bottom: bis-Et-SA+Zn^2+^). Figure S8: The Structural formula for intramolecular hydrogen bonds of bis-Et-SA. Figure S9. The ^13^C NMR spectroscopy of bis-Et-SA in CDCl_3._ Figure S10. The ^1^H NMR spectroscopy of bis-Et-4-NEt_2_ in CDCl_3_. Figure S11. The ^1^H NMR spectroscopy of bis-Et-5-NO_2_ in CDCl_3_. Figure S12. The ^13^C NMR spectroscopy of bis-Et-5-NO_2_ in CDCl_3_. Figure S13. The ^1^H NMR spectroscopy of bis-Et-Naph in DMSO-d_6_. Figure S14. The ^1^H NMR spectroscopy of bis-Ph-SA in CDCl_3_. Figure S15. The ^13^C NMR spectroscopy of bis-Ph-SA in CDCl_3_. Figure S16. The ^1^H NMR spectroscopy of bis-Ph-Naph in CDCl_3_. Figure S17. The ^1^H NMR spectroscopy of bis-CN-5-NO_2_ in CDCl_3_. Figure S18. The ^1^H NMR spectroscopy of bis-CN-Naph in CDCl_3_.
