# Supplementary material for: A Simple and Rapid “Turn-On” Fluorescent Probe Based on Binuclear Schiff Base for Zn2+ and Its Application in Cell Imaging and Test Strips
Source: Molecules. 2024 Dec 11;29(24):5850. doi: 10.3390/molecules29245850 (PMC11678835; doi:10.3390/molecules29245850)
Supplement: Supplementary file 1 [file molecules-29-05850-s001.zip › molecules-3309139-supplementary.pdf]

Supporting Information

A Simple and Rapid "Turn-On" Fluorescent Probe Based on Binuclear Schiff Base for Zn^{2+} and Its Application in Cell Imaging and Test Strips

Synthesis of Organic Dyes: 5,5'-Methylene-bis-salicylaldehyd was synthesized according to the literature [1]. The binuclear Schiff base ligands were prepared by a similar method according to previous reports [2–7]. The mixture of salicyaldehyde or salicylaldehyde derivatives (2.1 mmol) and the corresponding diamine (1.0 mmol) in 20 mL ethanol solution was refluxed at 78 °C for 5 h. After the reaction was complete, the mixture was cooled to 0 °C and then the product in crystal or powder was collected by filtration.

bis-Et-SA (48% yield): 1,2-ethylenediamine (0.60 g, 10 mmol) and salicylaldehyde (1.22 g, 10 mmol) were stirred for 12 h at 78℃ in 50 mL of absolute ethanol. The reaction is monitored by TLC (CH_2Cl_2). After the reaction is finished, it is placed in a mixture of ice water to cool the crystallization. A yellow-green solid precipitate was filtered and washed with ethanol and diethyl ether. The crude product was recrystallized in methanol to obtain Et-SA. Et-SA (0.32 g, 2 mmol) and 5,5'-Methylene-bis-salicylaldehyd (0.26 g, 1 mmol) were stirred for 18 h at 78℃ in 50 mL of absolute ethanol. The reaction is monitored by TLC (CH_2Cl_2:CH_3OH = 20:1). After the reaction is finished, it is placed in a mixture of ice water to recrystallization. A green solid precipitate was filtered and washed with ethanol and diethyl ether. The crude product was recrystallized in methanol to obtain bis-Et-SA. ^1H NMR (400 MHz, DMSO-d_6) δ 13.37 (s, 4H), 13.15 (s, 4H), 8.55 (m, 4H), 7.30 (m, 4H), 7.15 (dd, 4H, *J* 7.7 Hz, 1.7 Hz), 6.80 (d, 2H, *J* 8.3 Hz), 3.92 (s, 8H), 3.8(s, 2H). ^{13}C NMR (400 MHz, $CDCl_3$) δ 39.73, 59.79, 116.95, 117.06, 118.44, 118.67, 131.30, 131.38, 131.49, 132.39, 132.98, 159.39, 161.02, 166.48. Anal. Calcd. (Found): C, 72.24 (72.26), H, 5.88 (5.87), N, 10.21 (10.22), O, 11.66 (11.65). EI-MS, m/z 548.21 (calcd m/z = 548.24). m.p.100.1-100.2 ℃.

bis-Et-4-NEt_2 (53% yield): 1,2-ethylenediamine (0.60 g, 10 mmol) and 4-(diethylamino)salicylaldehyde (2.02 g, 10.5 mmol) were stirred for 12 h at 78℃ in 50 mL of absolute ethanol. The reaction is monitored by TLC (CH_2Cl_2). After the reaction is finished, it is placed in a mixture of ice water to cool the crystallization. A yellow solid precipitate was

filtered and washed with ethanol. The crude product was recrystallized in CH_2Cl_2 to obtain Et-4-NEt_2. Et-4-NEt_2 (0.46 g, 2 mmol) and 5,5'-Methylene-bis-salicylaldehyd (0.26 g, 1 mmol) were stirred for 18 h at 78℃ in 50 mL of absolute ethanol. The reaction is monitored by TLC (CH_2Cl_2:CH_3OH = 10:1). After the reaction is finished, it is placed in a mixture of ice water to recrystallization. A yellow solid precipitate was filtered and washed with ethanol and diethyl ether. The crude product was recrystallized in methanol to obtain bis-Et-4-NEt_2. ^1H NMR (400 MHz, DMSO-d_6) δ 10.91 (s, 4H), 9.85 (s, 4H), 8.53 (d, 4H, *J* 8.8 Hz), 7.19 (s, 2H), 7.17 (s, 2H), 6.95 (d, 2H, *J* 4.1 Hz), 6.78 (d, 2H, *J* 8.5 Hz), 4.06-3.76 (m, 10H), 3.42 (s, 8H), 1.22 (t, 12H, *J* 6.4 Hz). ^{13}C NMR (400 MHz, DMSO-d_6) δ 12.74, 39.73, 44.48, 58.26, 98.17, 102.47, 103.06, 108.35, 108.67, 131.41, 131.89, 132.99, 133.78, 151.51, 159.32, 164.38, 165.77. Anal. Calcd. (Found): C, 71.28 (71.26), H, 7.29 (7.30), N, 12.16 (12.17), O, 9.27 (9.27). EI-MS, m/z 690.31 (calcd m/z = 690.39). m.p.153.2-153.4 ℃.

bis-Et-5-NO_2 (40% yield): 1,2-ethylenediamine (0.60 g, 10 mmol) and 5-nitrosalicylaldehyde (1.67 g, 10 mmol) were stirred for 12 h at 78℃ in 50 mL of absolute ethanol. The reaction is monitored by TLC (CH_2Cl_2). After the reaction is finished, it is placed in a mixture of ice water. A yellow solid precipitate was filtered and washed with ethanol. The crude product was recrystallized in EtOAc to obtain Et-5-NO_2. Et-5-NO_2 (0.42 g, 2 mmol) and 5,5'-Methylene-bis-salicylaldehyd (0.26 g, 1 mmol) were stirred for 18 h at 78℃ in 50 mL of absolute ethanol. The reaction is monitored by TLC (CH_2Cl_2:CH_3OH = 10:1). After the reaction is finished, it is placed in a mixture of ice water. Remove the oily magazines and spin the clear liquid to 0℃ to precipitate the solid. A yellow solid precipitate was filtered and washed with ethanol and diethyl ether. The crude product was recrystallized in methanol to obtain bis-Et-5-NO_2. ^1H NMR (400 MHz, $CDCl_3$) δ 11.61 (s, 4H), 10.00 (s, 4H), 8.47 (s, 4H), 8.25 (s, 2H), 7.52 (s, 2H), 7.09-6.91 (m, 4H), 4.06 (s, 8H), 3.54 (s, 2H). ^{13}C NMR (400 MHz, $CDCl_3$) δ 39.73, 59.79, 116.95, 117.06, 118.44, 118.67, 131.30, 131.38, 131.49, 132.39, 132.98, 159.39, 161.02, 166.48. Anal. Calcd. (Found): C, 62.06 (62.05), H, 4.73 (4.74), N, 13.16 (13.17), O, 20.05 (20.04). EI-MS, m/z 638.27 (calcd m/z = 638.21). m.p.145.3-145.5 ℃.

bis-Et-Naph (32% yield): 1,2-ethylenediamine (0.60 g, 10 mmol) and 2-hydroxy-1-naphthalene formaldehyde (1.73g, 10 mmol) were stirred for 12 h at 78℃ in 50 mL of absolute ethanol. The reaction is monitored by TLC (CH_2Cl_2). After the reaction is finished, it is placed in a mixture of ice water. A yellow solid precipitate was filtered and washed with ethanol. The crude product was recrystallized in EtOAc to obtain Et-Naph. Et-Naph (0.43 g, 2 mmol) and 5,5'-Methylene-bis-salicylaldehyd (0.26 g, 1 mmol) were stirred for 18 h at 78℃

in 50 mL of absolute ethanol. The reaction is monitored by TLC (CH_2Cl_2:CH_3OH = 10:1). After the reaction is finished, it is placed in a mixture of ice water. Remove the oily magazines and spin the clear liquid to 0℃ to precipitate the solid. A yellow solid precipitate was filtered and washed with ethanol and diethyl ether. The crude product was recrystallized in methanol to obtain bis-Et-Naph. ^{1}H NMR (400 MHz, DMSO-d_6) δ 14.11 (s, 4H), 12.00 (s, 4H), 10.82 (s, 2H), 9.18 (d, 2H, *J* 8.6 Hz), 8.04 (d, 2H, *J* 8.4 Hz), 7.74 (d, 2H, *J* 9.4 Hz), 7.64 (d, 2H, *J* 7.9 Hz), 7.42 (t, 2H, *J* 7.7 Hz), 7.21 (t, 2H, *J* 7.4 Hz), 7.07 (d, 2H, *J* 8.9 Hz), 6.75 (d, 2H, *J* 9.3 Hz), 4.03 (s, 10H). ^{13}C NMR (400 MHz, DMSO-d_6) δ 39.42, 52.92, 114.73, 116.53, 118.47, 123.91, 126.65, 126.83, 128.08, 129.12, 130.12, 132.04, 132.41, 134.32, 134.35, 135.17, 163.52, 165.05, 168.32. Anal. Calcd. (Found): C, 75.91 (75.90), H, 5.59 (5.60), N, 8.64 (8.63), O, 9.86 (9.87). EI-MS, m/z 648.34 (calcd m/z = 648.27). m.p.123.1-123.2 ℃.

bis-Ph-SA (63% yield): 1,2-phenylenediamine (0.22 g, 2 mmol) and salicylaldehyde (0.24 g, 2 mmol) were stirred for 12 h at 78℃ in 30 mL of absolute ethanol. The reaction is monitored by TLC (CH_2Cl_2). After the reaction is finished, 5,5'-Methylene-bis-salicylaldehyd (0.26 g, 1 mmol) was added, the mixture stirred for 18 h at 78℃. The reaction is monitored by TLC (CH_2Cl_2:CH_3OH = 10:1). After the reaction is finished, it is placed in a mixture of ice water. Remove the oily magazines and spin the clear liquid to 0℃ to precipitate the solid. A yellow solid precipitate was filtered and washed with ethanol and diethyl ether. The crude product was recrystallized in CH_2Cl_2 to obtain bis-Ph-SA. ^{1}H NMR (400 MHz, $CDCl_3$) δ 12.93 (s, 4H), 10.93 (s, 4H), 8.81-8.17 (m, 4H), 7.44 (d, 4H, *J* 8.3 Hz), 7.34 (s, 4H), 7.22 (s, 4H), 7.15 (S, 2H), 7.06-6.92 (m, 4H), 3.94 (s, 2H). ^{13}C NMR (400 MHz, $CDCl_3$) δ 39.66, 117.66, 118.97, 119.08, 119.69, 127.67, 131.51, 132.18, 134.08, 142.57, 159.82, 161.36, 163.64. Anal. Calcd. (Found): C, 76.38 (76.37), H, 5.00 (5.01), N, 8.69 (8.70), O, 9.93 (9.92). EI-MS, m/z 644.21 (calcd m/z = 644.24). m.p.182.0-182.2 ℃.

bis-Ph-4-NEt_2 (47% yield): 1,2-phenylenediamine (1.08 g, 10 mmol) and 4-(diethylamino)salicylaldehyde (2.02 g, 10.5 mmol) were stirred for 12 h at 78℃ in 50 mL of absolute ethanol. The reaction is monitored by TLC (CH_2Cl_2). After the reaction is finished, it is placed in a mixture of ice water to crystallization. A orange-yellow solid precipitate was filtered and washed with ethanol. The crude product was recrystallized in CH_2Cl_2 to obtain Ph-4-NEt_2. Ph-4-NEt_2 (0.57 g, 2 mmol) and 5,5'-Methylene-bis-salicylaldehyd (0.26 g, 1 mmol) were stirred for 18 h at 78℃ in 50 mL of absolute ethanol. The reaction is monitored by TLC (CH_2Cl_2:CH_3OH = 10:1). After the reaction is finished, it is placed in a mixture of ice water to recrystallization. A orange-yellow solid precipitate was filtered and washed with ethanol and diethyl ether. The crude product was recrystallized in methyl alcohol to obtain

bis-Ph-4-NEt_2. ^{1}H NMR (400 MHz, DMSO-d_6) δ 12.89 (s, 4H), 10.26 (s, 4H), 8.72 (m, 2H), 7.82 (m, 6H), 7.33 (m, 6H), 6.04 (m, 6H), 3.88 (s, 2H), 3.37(q, 8H, *J* 7.1 Hz), 1.16(t,12H, *J* 6.8 Hz). ^{13}C NMR (400 MHz, DMSO-d_6) δ 12.99, 42.63, 46.42, 95.58, 102.40, 108.73, 117.67, 121.18, 121.76, 124.99, 130.83, 132.57, 133.75, 138.80, 142.78, 155.37, 161.55, 163.76, 167.84. Anal. Calcd. (Found): C, 74.78 (74.77), H, 6.40 (6.41), N, 10.68 (10.67), O, 8.14 (8.15). EI-MS, m/z 786.41 (calcd m/z = 786.39). m.p.193.1-193.3 ℃.

bis-Ph-5-NO_2 (43% yield): 1,2-phenylenediamine (1.08 g, 10 mmol) and 5-nitrosalicylaldehyde (1.67g, 10 mmol) were stirred for 12 h at 78℃ in 50 mL of absolute ethanol. The reaction is monitored by TLC (CH_2Cl_2). After the reaction is finished, it is placed in a mixture of ice water. A yellow solid precipitate was filtered and washed with ethanol. The crude product was recrystallized in CH_3OH to obtain Ph-5-NO_2. Ph-5-NO_2 (0.52 g, 2 mmol) and 5,5'-Methylene-bis-salicylaldehyd (0.26 g, 1 mmol) were stirred for 18 h at 78℃ in 50 mL of absolute ethanol. The reaction is monitored by TLC (CH_2Cl_2:CH_3OH = 10:1). After the reaction is finished, orange solid precipitate was filtered and washed with ethanol and diethyl ether. The crude product was recrystallized in methyl alcohol to obtain bis-Ph-5-NO_2**.** ^{1}H NMR (400 MHz, DMSO-d_6) δ 14.13 (s, 4H), 11.42(s, 4H), 10.31 (s, 2H), 9.14 (d, 2H, *J* 8.8), 8.40 (dd, 4H, *J* 19.2 Hz, 8.7 Hz), 8.28 (m, 4H), 8.20 (d, 2H, *J* 4.02 Hz), 8.0 (m,2H), 7.74 (m, 4H), 3.45(s, 2H). ^{13}C NMR (400 MHz, DMSO-d_6) δ 42.64, 117.67, 117.87, 121.18, 121.76, 122.51, 124.99, 126.60, 128.84, 130.83, 132.57, 138.80, 139.15, 142.78, 161.55, 167.91, 169.09. Anal. Calcd. (Found): C, 67.02 (67.03), H, 4.12 (4.11), N, 11.44 (11.43), O, 17.42 (17.43). EI-MS, m/z 734.24 (calcd m/z = 734.21). m.p.235.4-235.6 ℃.

bis-Ph-Naph (35% yield): 1,2-phenylenediamine (1.08 g, 10 mmol) and 2-hydroxy-1-naphthalene formaldehyde (1.73g, 10 mmol) were stirred for 12 h at 78℃ in 50 mL of absolute ethanol. The reaction is monitored by TLC (CH_2Cl_2). After the reaction is finished, it is placed in a mixture of ice water. A orange solid precipitate was filtered and washed with ethanol. The crude product was recrystallized in ethanol to obtain Ph-Naph. Ph-Naph (0.53 g, 2 mmol) and 5,5'-Methylene-bis-salicylaldehyd (0.26 g, 1 mmol) were stirred for 18 h at 78℃ in 50 mL of absolute ethanol. The reaction is monitored by TLC (CH_2Cl_2:CH_3OH = 10:1). After the reaction is finished, orange solid precipitate was filtered and washed with ethanol and diethyl ether. The crude product was recrystallized in methanol to obtain bis-Ph-Naph. ^{1}H NMR (400 MHz, $CDCl_3$) δ 10.92 (s, 4H), 9.85(s, 4H), 9.47 (s, 2H), 9.35 (s, 2H), 8.64 (s, 2H), 8.14(d, 4H, *J* 8.4 Hz), 7.83 (d, 4H, *J* 9.1 Hz), 7.74 (d, 4H, *J* 7.8 Hz), 7.52 (m, 2H), 7.43 (d, 4H, *J* 3.9 Hz), 7.36 (s, 2H), 3.95(s, 2H). ^{13}C NMR (400 MHz, DMSO-d_6) δ 42.65, 109.25, 117.67, 120.75, 121.18, 121.76, 125.01, 125.12, 128.42, 128.91, 128.99, 130.13, 130.83, 132.57,

136.82, 138.80, 142.78, 161.55, 161.87, 163.58, 167.91. Anal. Calcd. (Found): C, 75.91 (75.90); H, 5.59 (5.60); N, 8.64 (8.63), O, 9.86 (9.87). EI-MS, m/z 744.32 (calcd m/z = 744.27). m.p.178.6-178.8 ℃.

bis-CN-SA (63% yield): Dicyano-1,2-ethenediamine (1.08 g, 10 mmol) and salicylaldehyde (1.22 g, 10 mmol) were stirred for 12 h at 78℃ in 50 mL of absolute ethanol. The reaction is monitored by TLC (CH_2Cl_2). After the reaction is finished, brown solid precipitate was filtered and washed with ethanol and diethyl ether. The crude product was recrystallized in methanol to obtain CN-SA. CN-SA (0.42 g, 2 mmol) and 5,5'-Methylene-bis-salicylaldehyd (0.26 g, 1 mmol) were stirred for 18 h at 78℃ in 50 mL of absolute ethanol. The reaction is monitored by TLC (CH_2Cl_2:CH_3OH = 20:1). After the reaction is finished, brown solid precipitate was filtered and washed with ethanol and diethyl ether. The crude product was recrystallized in DMF/methanol to obtain bis-CN-SA. ^1H NMR (400 MHz, DMSO-d_6) δ 11.23 (s, 4H), 10.56 (s, 4H), 10.24 (d, 2H, *J* 6.4 Hz), 9.08 (m, 4H), 8.54 (m, 2H), 7.91 (d, 4H, *J* 7.0 Hz), 7.66 (d, 2H, *J* 7.7 Hz), 3.85(s, 2H). ^{13}C NMR (400 MHz, DMSO-d_6) δ 42.58, 114.71, 116.26, 117.66, 119.79, 124.81, 124.95, 125.68, 129.81, 133.03, 133.24, 134.77, 135.77, 157.51, 157.84, 161.24, 162.40. Anal. Calcd. (Found): C, 68.94 (68.93); H, 3.75 (3.74), N, 17.38 (17.39), O, 9.93 (9.94). EI-MS, m/z 644.24 (calcd m/z = 644.19). m.p.187.5-187.7 ℃.

bis-CN-4-NEt_2 (47% yield): Dicyano-1,2-ethenediamine (1.08 g, 10 mmol) and 4-(diethylamino)salicylaldehyde (2.02g, 10.5 mmol) were stirred for 12 h at 78℃ in 50 mL of absolute ethanol. The reaction is monitored by TLC (CH_2Cl_2). After the reaction is finished, tawny solid precipitate was filtered and washed with ethanol and diethyl ether. The crude product was recrystallized in DMF/methanol to obtain CN-4-NEt_2. CN-4-NEt_2 (0.57 g, 2 mmol) and 5,5'-Methylene-bis-salicylaldehyd (0.26 g, 1 mmol) were stirred for 18 h at 78℃ in 50 mL of absolute ethanol. The reaction is monitored by TLC (CH_2Cl_2:CH_3OH = 20:1). After the reaction is finished, violet solid precipitate was filtered and washed with ethanol and diethyl ether. The crude product was recrystallized in DMF/methanol to obtain bis-CN-4-NEt_2. ^1H NMR (400 MHz, DMSO-d_6) δ 11.12 (s, 4H), 10.26 (s, 4H), 8.70 (m, 4H), 7.63 (m, 4H), 7.34 (m, 2H), 6.95 (m, 2H), 3.90 (s, 2H), 3.43(q, 8H, *J* 7.1 Hz), 1.14(t, 12H, *J* 7.2 Hz). ^{13}C NMR (400 MHz, DMSO-d_6) δ 14.00, 42.56, 46.34, 102.76, 113.19, 114.71, 116.26, 117.66, 124.03, 124.95, 129.81, 132.20, 133.03, 135.77, 153.26, 157.51, 158.78, 160.27, 161.24. Anal. Calcd. (Found): C, 68.69 (68.70), H, 5.38 (5.39), N, 17.80 (17.79), O, 8.13 (8.12). EI-MS, m/z 786.37 (calcd m/z = 786.34). m.p.226.2-226.5℃.

bis-CN-5-NO_2 (40% yield): Dicyano-1,2-ethenediamine (1.08 g, 10 mmol) and 5-nitrosalicylaldehyde (1.67g, 10 mmol) were stirred for 12 h at 78℃ in 50 mL of absolute ethanol. The reaction is monitored by TLC (CH_2Cl_2). After the reaction is finished, it is placed in a mixture of ice water. A red solid precipitate was filtered and washed with ethanol. The crude product was recrystallized in CH_3OH to obtain CN-5-NO_2. CN-5-NO_2 (0.53 g, 2 mmol) and 5,5'-Methylene-bis-salicylaldehyd (0.26 g, 1 mmol) were stirred for 18 h at 78℃ in 50 mL of absolute ethanol. The reaction is monitored by TLC (CH_2Cl_2:CH_3OH = 10:1). After the reaction is finished, deep red solid precipitate was filtered and washed with ethanol and diethyl ether. The crude product was recrystallized in DMF/CH_3OH to obtain bis-Ph-5-NO_2. ^{1}H NMR (400 MHz, $CDCl_3$) δ 10.94 (s, 4H), 9.87(s, 4H), 8.96 (s, 2H), 8.88 (s, 2H), 8.83 (s, 2H), 8.50 (s, 2H), 7.05 (d, 2H, *J* 8.7 Hz), 7.00-6.97 (m, 2H) 3.96(s, 2H). ^{13}C NMR (400 MHz, DMSO-d_6) δ 42.57, 114.71, 114.76, 115.76, 116.26, 117.66, 119.42, 123.52, 124.86, 124.95, 127.70, 129.81, 133.03, 135.77, 142.93, 152.91, 157.51, 161.24, 165.23. Anal. Calcd. (Found): C, 60.49 (60.48); H, 3.02 (3.03); N, 19.07 (19.06), O, 17.42 (17.43). EI-MS, m/z 734.10 (calcd m/z = 734.16). m.p.232.4-232.6 ℃.

bis-CN-Naph (32% yield): Dicyano-1,2-ethenediamine (1.08 g, 10 mmol) and 2-hydroxy-1-naphthalene formaldehyde (1.73g, 10 mmol) were stirred for 12 h at 78℃ in 50 mL of absolute ethanol. The reaction is monitored by TLC (CH_2Cl_2). After the reaction is finished, it is placed in a mixture of ice water. A orange red precipitate was filtered and washed with ethanol. The crude product was recrystallized in ethanol to obtain CN-Naph. CN-Naph (0.53 g, 2 mmol) and 5,5'-Methylene-bis-salicylaldehyd (0.26 g, 1 mmol) were stirred for 18 h at 78℃ in 50 mL of absolute ethanol. The reaction is monitored by TLC (CH_2Cl_2:CH_3OH = 10:1). After the reaction is finished, deep red solid precipitate was filtered and washed with ethanol and diethyl ether. The crude product was recrystallized in methanol to obtain bis-CN-Naph. ^{1}H NMR (400 MHz, $CDCl_3$) δ 10.94 (s, 2H), 9.87(s, 2H), 8.90 (s, 4H), 7.55(d, 2H, *J* 7.9 Hz), 7.52 (d, 2H, *J* 1.7 Hz), 7.50 (d, 2H, *J* 1.6 Hz), 7.48 (s, 2H), 7.10 (d, 4H, *J* 8.4 Hz), 7.04 (s, 4H), 6.99 (d, 2H, *J* 4.8 Hz), 3.96(s, 2H). ^{13}C NMR (400 MHz, DMSO-d_6) δ 42.59, 114.29, 114.71, 115.88, 116.26, 117.41, 117.66, 118.81, 124.95, 125.45, 129.81, 130.43, 130.86, 130.91, 131.19, 131.50, 133.03, 165.23, 161.24, 157.51, 152.91, 142.93, 135.77, 133.03, 135.15, 135.77, 154.53, 157.51, 160.14, 161.24. Anal. Calcd. (Found): C, 72.57 (72.58); H, 3.79 (3.78); N, 15.05 (15.04), O, 8.59 (8.60). EI-MS, m/z 744.27 (calcd m/z = 744.22). m.p.257.3-257.5 ℃.

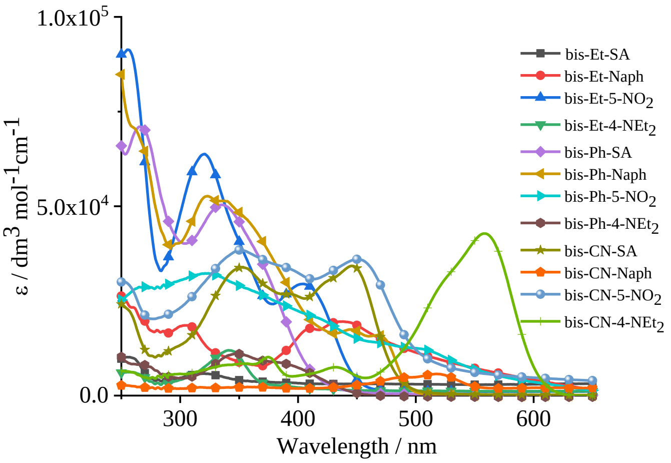

Figure S1. Absorption spectra of all binuclear Schiff base ligands in MeCN at room temperature.

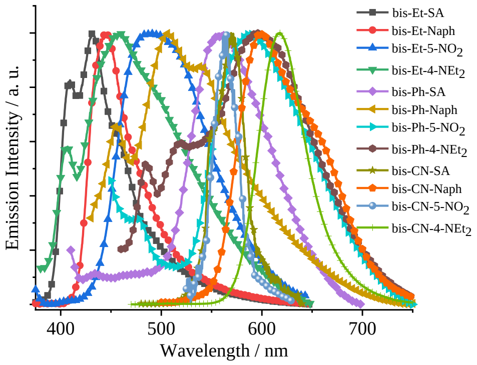

Figure S2. Normalized emission spectra of all the bis-Schiff ligands in MeCN at room temperature.

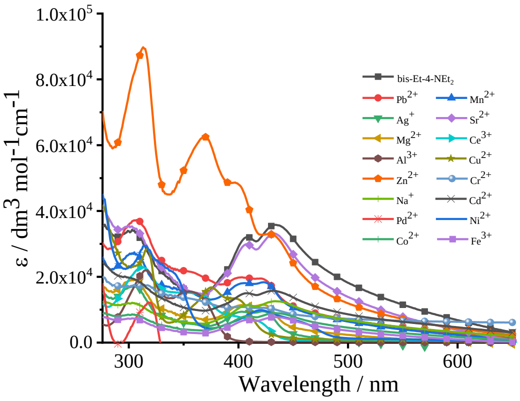

Figure S3. Absorption spectra of bis-Et-4-NEt₂ (1.0 × 10^{-5} mol dm^{-3} in MeCN) upon the addition of 2 equiv. of different metal ions.

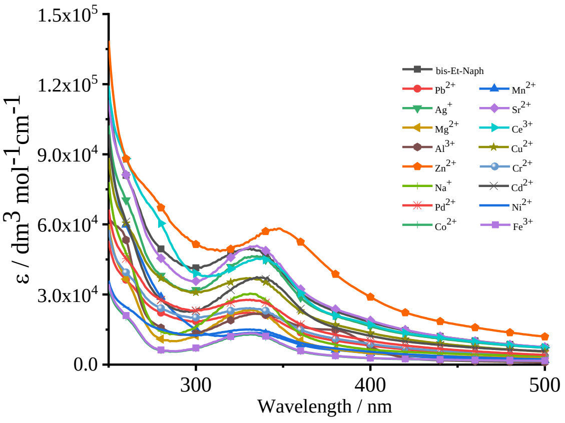

Figure S4. Absorption spectra of bis-Et-Naph (1.0×10^{-5} mol dm^{-3} in MeCN) upon the addition of 2 equiv. of different metal ions.

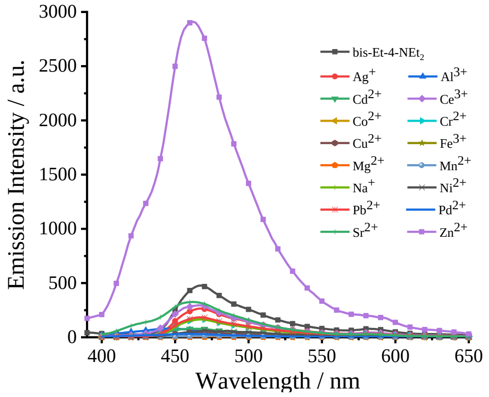

Figure S5. The emission spectra of bis-Et-4-NEt$_2$ (1.0×10^{-5} mol dm^{-3} in MeCN, excited at 370 nm) upon the addition of 2 equiv. of different metal ions.

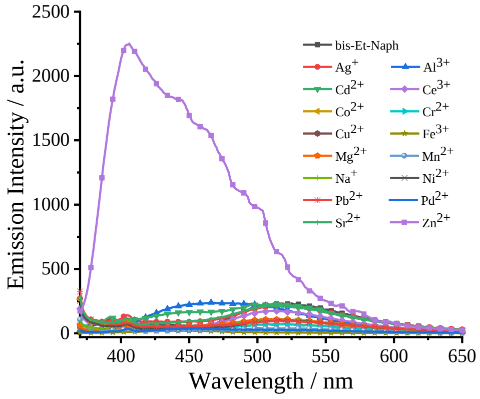

Figure S6. The emission spectra of bis-Et-Naph (1.0×10^{-5} mol dm^{-3} in MeCN, excited at 360 nm) upon the addition of 2 equiv. of different metal ions.

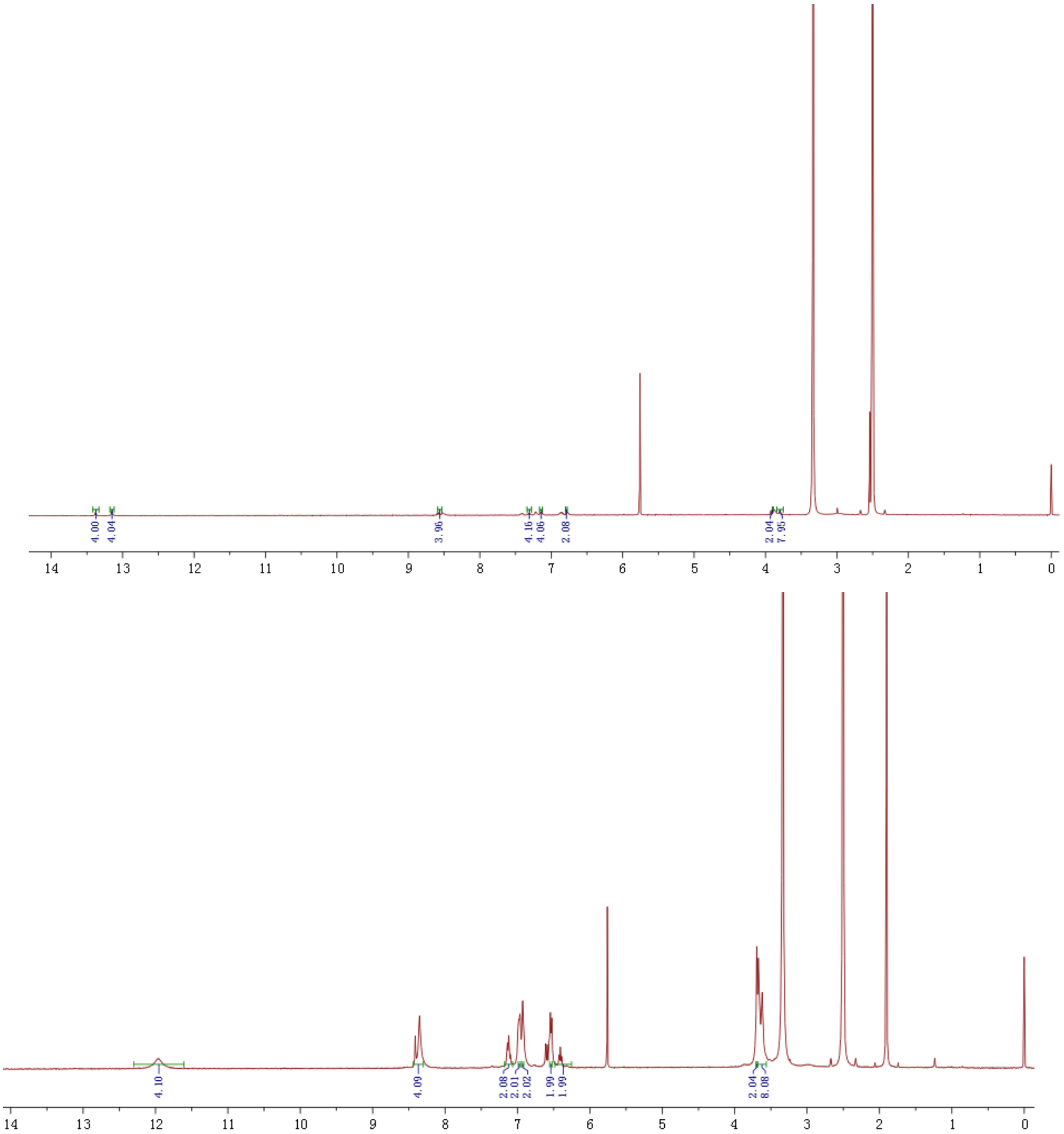

Figure S7. The ^{1}H NMR spectroscopy of bis-Et-SA and bis-Et-SA+Zn^{2+} in DMSO-d_6 (Top: bis-Et-SA. Bottom: bis-Et-SA+Zn^{2+}).

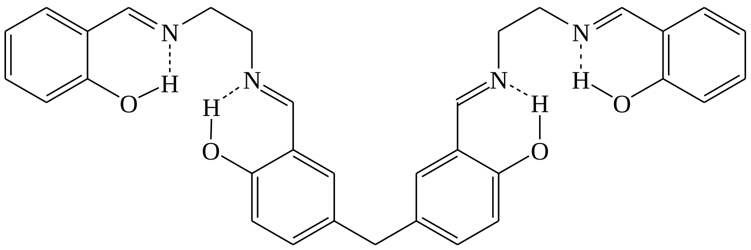

Figure S8. The Structural formula for intramolecular hydrogen bonds of bis-Et-SA.

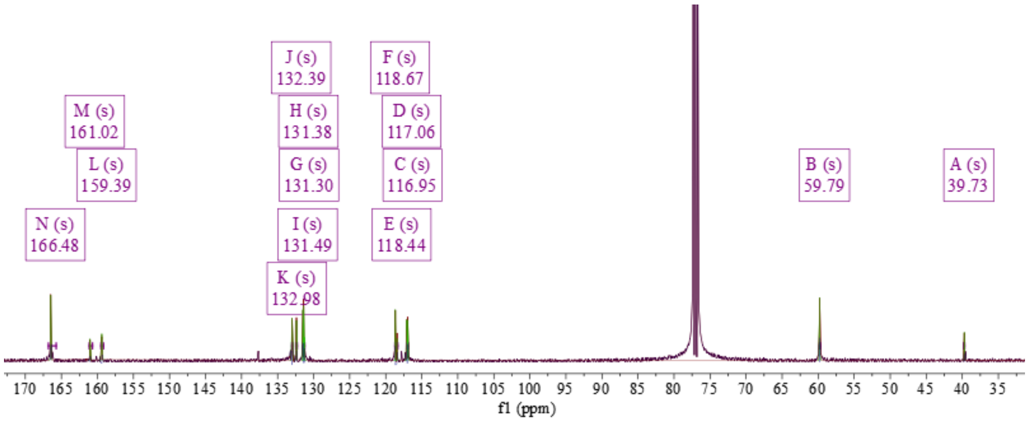

Figure S9. The ^{13}C NMR spectroscopy of bis-Et-SA in $CDCl_3$.

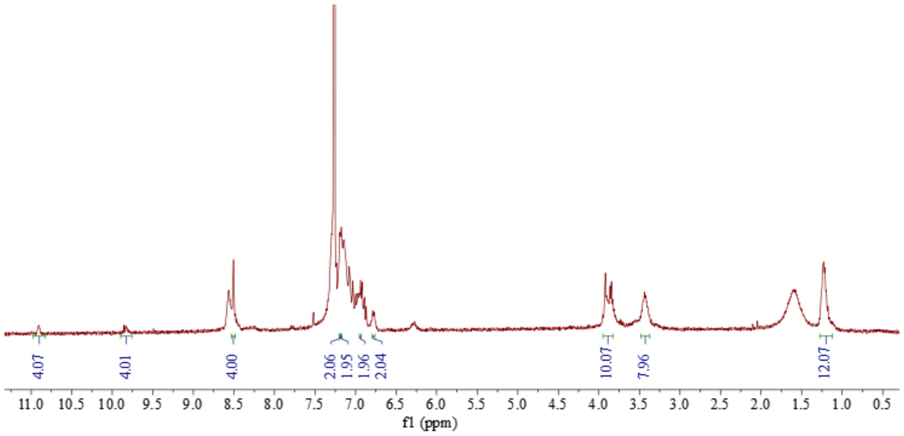

Figure S10. The ^{1}H NMR spectroscopy of bis-Et-4-NEt_2 in $CDCl_3$.

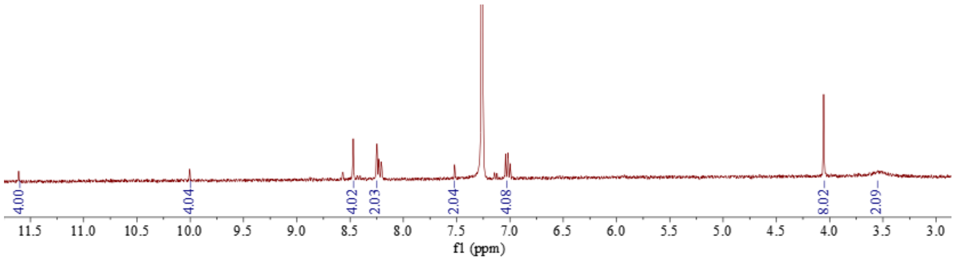

Figure S11. The ^{1}H NMR spectroscopy of bis-Et-5-NO_2 in $CDCl_3$.

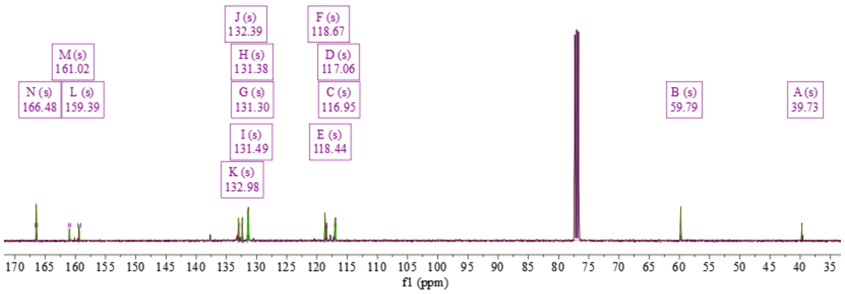

Figure S12. The ^{13}C NMR spectroscopy of bis-Et-5-NO_2 in $CDCl_3$.

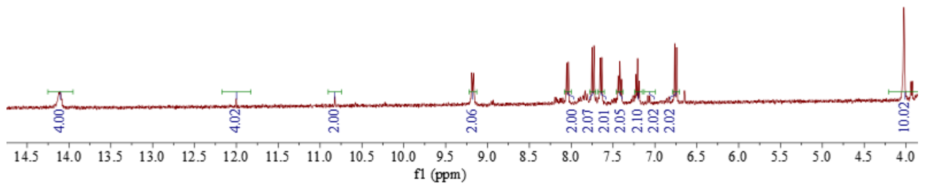

Figure S13. The ^{1}H NMR spectroscopy of bis-Et-Naph in DMSO-d_6.

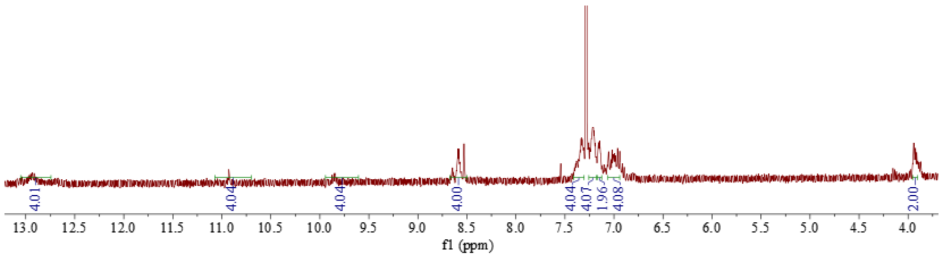

Figure S14. The ^{1}H NMR spectroscopy of bis-Ph-SA in $CDCl_3$.

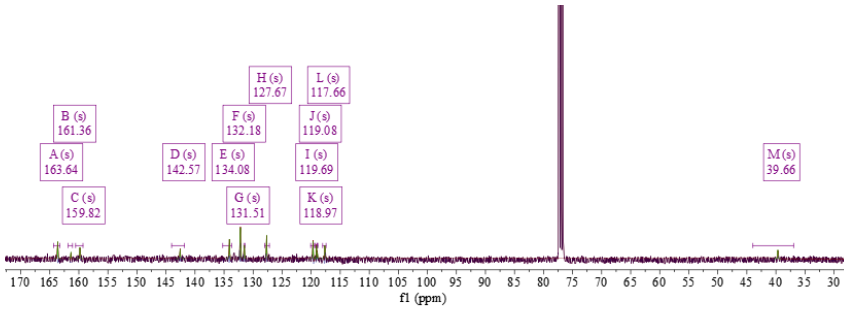

Figure S15. The ^{13}C NMR spectroscopy of bis-Ph-SA in $CDCl_3$.

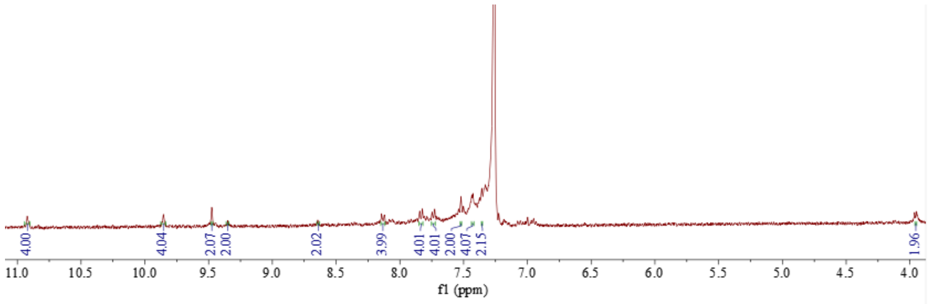

Figure S16. The ^{1}H NMR spectroscopy of bis-Ph-Naph in $CDCl_3$.

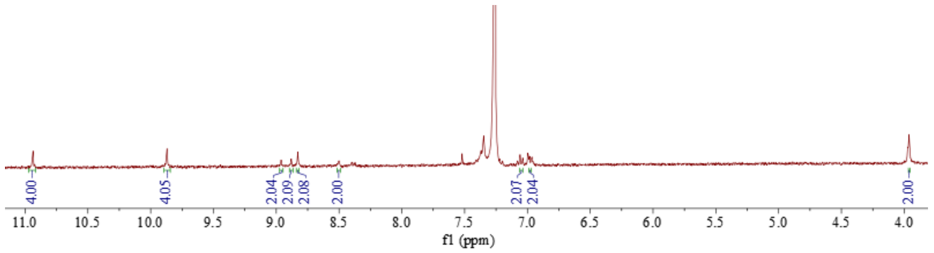

Figure S17. The ^{1}H NMR spectroscopy of bis-CN-5-NO_2 in $CDCl_3$.

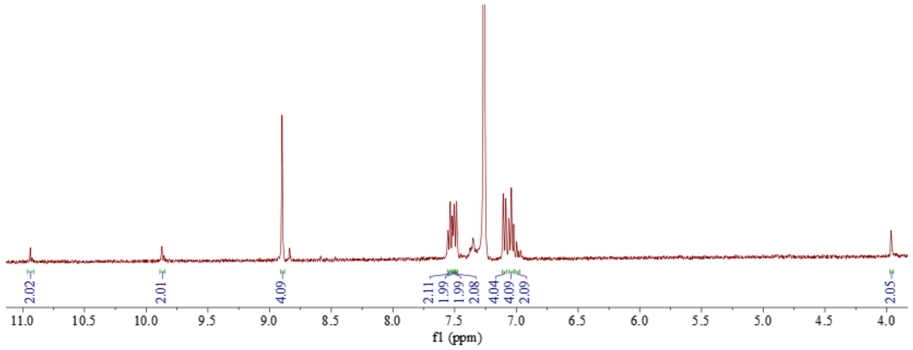

Figure S18. The ^{1}H NMR spectroscopy of bis-CN-Naph in $CDCl_3$.

References

1. Marvel, C.S.; Tarköy, N. Heat Stability Studies on Chelates from Schiff Bases of Salicylaldehyde Derivatives. *J. Am. Chem. Soc.* **1957**, *79*, 6000–6002.
2. Zhou, L.; Cai, P.Y.; Feng, Y.; Cheng, J.H.; Xiang, H.F.; Liu, J.; Wu, D.; Zhou, X.G. Synthesis and photophysical properties of water-soluble sulfonato-Salen-type Schiff bases and their applications of fluorescence sensors for Cu^{2+} in water andliving cells. *Anal. Chim. Acta* **2012**, *735*, 96–106.
3. Wang, M.; Cheng, C.; Song, J.; Wang, J.; Zhou, X.; Xiang, H.; Liu, J. Multiple Hydrogen Bonds Promoted ESIPT and AIE-active Chiral Salicylaldehyde Hydrazide. *J. Chin. J. Chem.* **2018**, *36*, 698–707.
4. Cheng, J.H.; Wei, K.Y.; Ma, X.F.; Zhou, X.G.; Xiang, H.F. Synthesis and Photophysical Properties of Colorful Salen-Type Schiff Bases. *J. Phys. Chem. C* **2013**, *117*, 16552–16563.
5. Cheng, J.H.; Li, Y.X.; Sun, R.; Liu, J.Y.; Gou, F.; Zhou, X.G.; Xiang, H.F.; Liu, J. Functionalized Salen ligands linking with non-conjugated bridges: Unique and colorful aggregation-induced emission, mechanism, and applications. *J. Mater. Chem. C* **2015**, *3*, 11099–11110.
6. Shimakoshi, H.; Ninomiya, W.; Hisaeda, Y. Reductive coupling of benzyl bromide catalyzed by a novel dicobalt complex having two salen units. *J. Chem. Soc. Dalton Trans.* **2001**, *13*, 1971–1974.
7. Shimakoshi, H.; Goto, A.; Tachi, Y.; Naruta, Y.; Hisaeda, Y. Synthesis and redox behavior of dialkylated dicobalt complexes having two discrete salen units. *Tetrahedron Lett.* **2001**, *42*, 1949–1951.
